# Testing methods and physical qualities of male age grade rugby union players: A systematic review

**DOI:** 10.1371/journal.pone.0233796

**Published:** 2020-06-04

**Authors:** Cameron Owen, Kevin Till, Jonathon Weakley, Ben Jones

**Affiliations:** 1 Leeds Beckett University, Carnegie Applied Rugby Research (CARR) centre, Carnegie School of Sport, Leeds, United Kingdom; 2 Yorkshire Carnegie Rugby Union club, Leeds, United Kingdom; 3 Leeds Rhinos Rugby League club, Leeds, United Kingdom; 4 School of Behavioural and Health Sciences, Australian Catholic University, Brisbane, Queensland, Australia; 5 England Performance Unit, The Rugby Football League, Leeds, United Kingdom; 6 School of Science and Technology, University of New England, Armidale, NSW, Australia; 7 Division of Exercise Science and Sports Medicine, Department of Human Biology, Faculty of Health Sciences, the University of Cape Town and the Sports Science Institute of South Africa, Cape Town, South Africa; University of Cassino e Lazio Meridionale, ITALY

## Abstract

**Background:**

Rugby union match demands are complex, requiring the development of multiple physical qualities concurrently. Quantifying the physical qualities of age grade rugby union players is vital for practitioners to support athlete preparation and long-term development.

**Aim:**

This systematic review aimed to identify the methods used to quantify the physical qualities of male age grade (≤ Under-20) rugby union players, present the normative values for physical qualities, and compare physical qualities between age grades and positions.

**Methods:**

Electronic databases were systematically reviewed from the earliest record to November 2019 using key words relating to sex, age, sport and physical testing.

**Results:**

Forty-two studies evaluated the physical qualities of age grade rugby union players. Seventy-five tests were used to quantify body composition, muscular strength, muscular power, linear speed, change of direction ability, aerobic capacity and anaerobic endurance. Thirty-one studies met the eligibility criteria to present the physical qualities. Physical qualities differentiate between age groups below Under-16, while differences in older age groups (Under-16 to Under-20) are not clear. Positional differences are present with forwards possessing greater height, body mass, body fat percentage and strength while backs are faster and have greater aerobic capacities.

**Conclusions:**

A wide variety of tests are used to assess physical qualities limiting between study comparisons. Although differences in older age grades are unclear, older age groups (Under-19-20) generally performed better in physical tests. Positional differences are associated with match demands where forwards are exposed to less running but a greater number of collisions. Practitioners can use the results from this review to evaluate the physical qualities of age grade rugby union players to enhance training prescription, goal setting and player development. Future research should consider the use of national standardised testing batteries due to the inconsistency in testing methods and small samples limiting the reporting of positional differences.

## Introduction

Rugby union (RU) is a sport played in 121 countries, with over 8.5 million participants [1]. Professional competition is mainly based within Tier 1 nations from the southern hemispheres Rugby Championship (Argentina, Australia, New Zealand and South Africa) and the northern hemispheres Six Nations (England, France, Ireland, Italy, Scotland and Wales). During a senior level match, two teams of 15 players (with a maximum of 8 replacements totalling a 23 man squad) compete over two 40-minute halves with half time not exceeding 15 minutes [2]. Playing positions can be categorised into two positional groups, forwards (prop, hooker, lock, flanker and no. 8) and backs (scrum half, fly-half, centre, wing and full back) [3]. Specialist roles during match play determine the demands for each position with the forwards suggested to be ball winners while the backs are ball carriers [4].

To support the development of young RU players towards the senior game, many national governing bodies have established age grade development pathways culminating with the Under 20 (U20) World Championship. Developing technical, tactical and physical qualities throughout the pathway is key to enhancing RU performance [5–8]. RU match play is complex, with collisions, high speed running, and technical elements being interspersed with periods of recovery [9,10]. The match demands of RU players do not only vary depending on playing position but also age and playing level [11,12]. Over the course of an U20 International game forwards and backs are required to run ~5370 and ~6230 m, with ~284 and ~657 m covered at high speed (> 5.0 m·s^-1^), respectively [13]. To date there is no information regarding the velocities achieved during U20 International games however both age grade and senior players achieve velocities greater than 90% of their maximum sprint speed during match-play [14,15]. A similar number of collisions has also been observed across playing levels for both forwards (~40) and backs (~13), although the magnitude of collisions may differ due to differences in body mass [16,17]. Due to the complex and dynamic nature of RU multiple physical qualities need to be developed to optimise RU performance.

Although the physical qualities of senior RU players have previously been reviewed [10], only the height and body mass of age grade RU players are summarised in the current literature [18]. Throughout the development pathway it is important for practitioners to appropriately develop the physical qualities of players to promote optimum performance and long-term athletic development. Duthie [19] provided a framework for the development of physical qualities of elite RU players which includes; needs analysis, evaluation of the athlete, prescription of an intervention and the evaluation of progress. Effective evaluation of young athletes is important for both training prescription and both long- and short-term goal setting. Team coaches, sports scientists and strength and conditioning coaches can use objective markers in combination with statistical methods (e.g. z-scores) to evaluate athlete performance, inform talent identification and guide physical development [20]. It would therefore be beneficial to collate the findings from previous research to provide an understanding of the development of physical qualities in age grade RU players.

When quantifying the physical qualities of RU players practitioners have a plethora of testing methods and variables to choose from. Unlike sports such as the Australian Football League (AFL) which utilise a physical testing combine, both researchers [10,21] and practitioners [22,23] employ a variety of testing methods in the rugby codes. Variation in testing methods can prove challenging in both research and practice when attempting to understand the required physical qualities of developing athletes [24]. A standardised testing battery, such as a combine, increases test homogeneity in the research allowing for comparisons across the participation pathway [24]. Although Till et al. [25] only found a variation in methods quantifying body composition and aerobic capacity when reviewing the physical qualities of age grade Rugby League (RL) players the review was not systematic and only papers produced by six lead authors were reported, potentially limiting the testing methods observed. Furthermore, no exclusion criteria or rationale was provided concerning the testing methods reported, and consequently alternative methods used within the sport are not discussed. Utilising a systematic approach to identify all testing methods used within the literature will not only provide a consensus on the most common physical tests used within age grade RU but also rationalise the selection of methods reported when collating the findings on the physical qualities of age grade RU players.

The purpose of this systematic review is twofold. Firstly, to identify the tests used to measure the physical qualities of age grade (≤ U20) RU players and secondly to present and compare the differences of physical qualities between age groups and playing positions. The review will provide normative values for the physical qualities of age grade rugby players enhancing the ability of practitioners to evaluate physical testing data and prescribe training, thus optimising rugby performance and long-term athlete development.

## Methods

### Design and search strategy

A systematic review was performed in accordance with the Preferred Reporting Items of Systematic Reviews and Meta-analyses (PRISMA) statement [26], with the exception of preregistration (S1 File). A search of databases (MEDLINE, PubMed, CINAHL, SPORT Discuss and SCOPUS) was conducted for papers published from the earliest record to November 2019. Key words were identified to define sex, age, sport and physical testing (Table 1) for the search, which were linked using Boolean terms. In addition to the systematic search, reference lists of selected papers were reviewed for potentially eligible papers.

**Table 1:** Search strategy terms.

Search 1 (sex)	Search 2 (age)	Search 3 (sport)	Search 4 (physical testing)
NOT female	Adolescents OR youth OR teenagers OR student OR junior OR academy OR ‘young adult’	‘Rugby union’ OR rugby OR football NOT soccer OR League OR ‘gaelic football’	‘Fitness testing’ OR ‘physical characteristics’ OR ‘physical qualities’ OR ‘physical performance’ OR ‘physical profile’ OR anthropometric OR ‘body height’ OR ‘body weight’ OR skinfold OR ‘body composition’ OR ‘body fat’ OR power OR ‘countermovement jump’ OR ‘vertical jump’ OR ‘muscular strength’ OR acceleration OR speed OR sprint OR running OR agility OR ‘change of direction’ OR fitness OR ‘physical fitness’ OR ‘aerobic capacity’ OR ‘cardiorespiratory fitness’ OR ‘repeated-sprint ability’ OR ‘anaerobic’

### Study selection

After removing duplicates, two reviewers (CO, JW) independently screened the titles and abstracts for eligibility against the criteria. Conflicts were resolved through discussion, or a third reviewer if required. The full text of articles that were not excluded during this process were then reviewed. The authors of each article were not blinded to the reviewers.

To address the first research aim, studies were eligible for inclusion if they explored the physical qualities (anthropometrics, strength, power, speed, change of direction, aerobic capacity and anaerobic endurance) of male, age grade (≤ U20) RU players. The article was included if it identified at least one anthropometric or physical quality and this was the primary aim of the paper. Identification of these qualities as a result of other research aims (e.g. match demands, fatigue and nutrition) resulted in exclusion. Age was identified by either the reported age or the age grade stated in the article. Only articles written in English that appeared in a peer reviewed journal were included. If further information was required regarding the study, such as the age grade of players, the corresponding author was contacted and if there was no response the article was excluded.

For the second aim, common tests were identified for study inclusion and only studies that clearly identified the physical quality of a single age grade (e.g. U13 or U16) and not spanning a range (e.g. U17-U20) were included. If the study did not report team or positional means and reported multiple groups as part of an intervention it was excluded.

### Assessment of methodological quality

The Downs & Black [27] assessment scale was modified to review the methodological quality of included articles. Due to the cross-sectional nature of the data extracted, similarly to previous research [28], only 12 questions (1–4, 6, 7, 10–12, 16, 18, 20) were used which were logically relevant. Due to no intervention taking place question 4 was interpreted as “are the tests in the study clearly described”. The reporting of effect sizes was deemed acceptable for question 10. Failure to meet the criteria resulted in a score of “0” with sufficient information resulting in a score of “1”. No studies were removed as a result of poor methodological quality.

### Data extraction

The initial extraction withdrew publication data (authors, year of publication and sample information), all tests performed to quantify anthropometrics or physical qualities (e.g., one-repetition maximum (RM) back squat, countermovement jump, 10m sprint), and output measures reported (e.g., body fat percentage, jump height, sprint time). If the methods of physical tests could not be identified from the article or refences provided the tests were not included in the extraction.

Following the extraction of the tests used, data relating to the age grade, level of competition and playing position of the participants were noted along with results for anthropometrical (height, weight and body composition), strength (bench press and squat variations), power (vertical jump height, countermovement jump height and peak power), speed (10m, 20m, 30m and 40m), change of direction (Illinois agility test, T-test, pro agility and 505) and aerobic capacity tests (multistage fitness test (MSFT), Yo-Yo endurance test level 1 (YYE1), Yo-Yo intermittent recovery test level 1 (YYIR1) and 30–15 intermittent fitness test (30-15IFT)). When needed WebPlotDigitiser V4.1 was used to extract means and measures of variance (standard deviations or confidence intervals) from figures [29]. When cross-sectional data for a single age group was presented for multiple years, the most recent time point was used. In the case of intervention studies, the baseline score was extracted to remove bias of the intervention.

### Statistical analysis

The data are reported as a mean with a measure of variance as provided in the article. No further statistical analysis was carried out on the data.

## Results

### Identification and selection of studies

**Fig 1:**
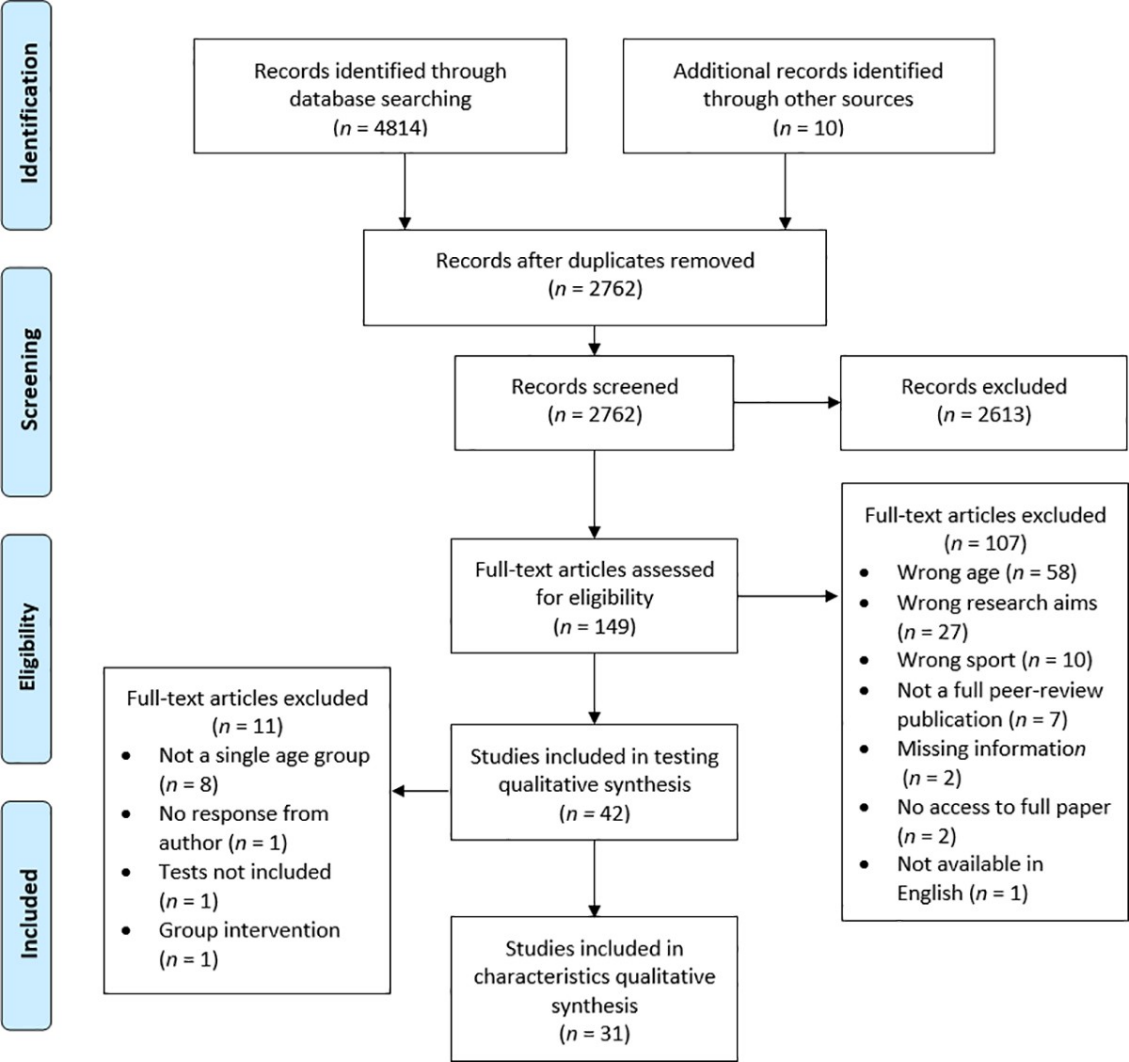
Flow of selection process of eligible studies for qualitative synthesis.

The search identified 4,814 articles with a further 10 identified through hand searching reference lists. Following the removal of duplicates, 2,762 were screened and 149 studies were reviewed in detail (Fig 1). The initial extraction process found 42 studies assessing the physical qualities of age grade RU players with 31 studies used to present the physical qualities by age grade and position.

### Study characteristics

Table 2 shows the characteristics of the studies that were included within the systematic review. Sample size ranged from 15–4007 with a median of 83 participants and the number of teams used to recruit the sample ranged from 1–188 with a median of 1. The age range of participants was U11 to U20 from an array of playing levels; school, community, club, representative, provincial, academy, national draft camp, national and university. A cross sectional study design was used by thirty-three (78%) studies, with six (14%) studies using an experimental design and three (7%) studies using a longitudinal design. Research was conducted in 11 countries; Australia, Ireland, South Africa, England, Scotland, Wales, Zimbabwe, Portugal, Italy, Brazil and France.

**Table 2:** Characteristics of the studies included in the review.

Author	Number of participants	Number of teams	Playing standard	Age (years)	Study design	Country
Argus et al. [58]	51	1	Academy and school	Academy; 19.6 ± 1.8 High school; 16.6 ± 0.8	Cross-sectional	New Zealand
Ball et al. [30]	51	1	University	U20; 19.2 ± 0.7	Cross-sectional	Australia
Barr et al. [71]	31	1	National	U20; 19.2 ± 0.9	Cross-sectional	Australia
Casserly et al. [68]	15	1	Academy	U18-U20	Longitudinal	Ireland
Chiwaridzo et al. [37]	71	2	School	U16 elite school; 14.9 ± 0.31 U16 sub-elite school; 14.8 ± 0.43	Cross-sectional	Zimbabwe
Chiwaridzo et al. [38]	87	2	School	U19 elite school; 17.5 ± 0.85 U19 sub-elite school; 17.4 ± 0.87	Cross-sectional	Zimbabwe
Darrall-Jones et al. [39]	80	1	Academy	U16 backs; 15.6 ± 0.2 U16 forwards; 15.4 ± 0.3 U18 backs; 16.9 ± 0.6 U18 forwards; 16.9 ± 0.5	Cross-sectional	England
Darrall Jones et al. [40]	75	1	Academy	U16;15.2 ± 2.3 U18;17.2 ± 0.6	Cross-sectional	England
Darrall Jones et al. [73]	53	1	Academy	U16; 15.5 ± 0.3 U18; 16.9 ± 0.5	Cross-sectional	England
De la Port & Spamer [31]	150	1	National	U16 and U18	Longitudinal	South Africa
Delahunt et al. [54]	136	5	School	U18; 16.93 ± 0.87	Cross-sectional	Ireland
Durandt et al. [74]	4007	188	Provincial	U18	Cross-sectional	South Africa
Durandt et al. [32]	174	1	National	U16 and U18	Cross-sectional	South Africa
Fontana et al. [47]	531	1	National draft camp	U16	Cross-sectional	Italy
Grobler et al. [35]	213	6	School	U14, U15 and U16	Cross-sectional	South Africa
Harries et al. [53]	26	2	Representative	14–18	Quasi-experimental	Australia
Harries et al. [51]	16	1	Representative	15–18	Randomised controlled trial	Australia
Howard et al. [69]	51	1	Academy	15.9 ± 0.7	Cross-sectional	England
Jones et al. [59]	184	5	School and academy	U18 school; 17.3 ± 0.6 U18 Academy; 17.5 ± 0.6	Cross-sectional	England
Kobal et al. [64]	88	1	Club	U15, U17 and U19	Cross-sectional	Brazil
Krause et al. [65]	485	NA	Community	U12, U13, U14 and U15	Cross-sectional	Australia
Lombard et al. [57]	453	1	National	U20; 18.1 ± 0.7	Repeated cross-sectional	South Africa
Nutton et al. [62]	472	NA	School	12–18 years	Cross-sectional	Scotland
Parsonage et al. [66]	156	4	Academy	U16	Cross-sectional	Wales
Pienaar & Spamer [43]	31	NA	Provincial	U11	Longitudinal	South Africa
Pienaar et al. [46]	45	3	School	U11	Cross-sectional	South Africa
Pienaar & Coetzee [48]	40	1	University	U19; 18.9 ± 0.4	Randomised controlled trial	South Africa
Plotz [63]	64	3	School and provincial	U18	Cross-sectional	South Africa
Sedeaud [104]	448	16	Club	U15	Cross-sectional	France
Smart & Gill [41]	44	NA	Provincial	U14 –U18	Pre-post measures	New Zealand
Spamer [50]	382	NA	National, provincial and school	U12	Cross-sectional	South Africa
Spamer & Hattingh [49]	331	2	School (U15 & U18) and club (U19 & U20)	U15, U18, U19 and U20	Cross-sectional	South Africa
Spamer & Winsley [44]	83	2	School	U12	Cross-Sectional	England
Spamer & Winsley [45]	NA	3	School and provincial	U18	Cross-sectional	England/ South Africa
Spamer & De la Port [33]	146	1	National	U16 and U18	Cross-sectional	South Africa
Spamer et al. [42]	8	3	Provincial	U16	Cross-sectional	New Zealand/ South Africa
Speirs et al. [61]	18	1	Academy	18.1 ± 0.5	2 x 2 mixed	Scotland
Van Gent & Spamer [34]	80	1	Provincial	U13, U16, U18 and U19	Cross-sectional	South Africa
Vaz et al. [36]	41	1	National	U19	Cross-sectional	Portugal
Walsh et al. [52]	203	6	School	15–18	Cross-sectional	Ireland
Weakley et al. [60]	35	4	School	U18 (16.9 ± 0.4)	Pre-post measures	England
Wood et al. [67]	89	2	National	18.66 ± 0.58	Cross-sectional	Ireland

Data are expressed as mean ± SD

### Methodological quality

The score from the assessment of methodological quality can be observed in Table 3, ranging from 5–10 for the 12 items assessed.

**Table 3:** Methodological quality assessment (Downs and Black [27]).

Study	Question number												Total score
	1	2	3	4	6	7	10	11	12	16	18	20	
Argus et al. [58]	1	1	1	1	1	1	0	0	0	1	1	1	9
Ball et al. [30]	1	1	1	1	1	1	1	0	0	1	1	1	10
Barr et al. [71]	1	1	1	1	1	1	1	0	0	1	1	0	9
Casserly et al. [68]	1	1	1	1	1	1	1	0	0	1	1	1	10
Chiwaridzo et al. [37]	1	1	1	1	1	1	1	0	0	1	1	1	10
Chiwaridzo et al. [38]	1	1	1	1	1	1	1	0	0	1	1	1	10
Darrall-Jones et al. [39]	1	1	1	1	1	1	1	0	0	1	1	1	10
Darrall Jones et al. [40]	1	1	1	1	1	1	1	0	0	1	1	1	10
Darrall Jones et al. [73]	1	1	1	1	1	1	1	0	0	1	1	1	10
De la Port & Spamer [31]	1	1	1	0	1	1	1	0	0	1	1	0	8
Delahunt et al. [54]	1	1	1	1	1	1	0	0	0	1	1	1	9
Durandt et al. [74]	1	1	1	1	1	1	1	0	0	1	1	1	10
Durandt et al. [32]	1	1	1	1	1	1	1	0	0	1	1	0	9
Fontana et al. [47]	1	1	1	1	1	1	0	0	0	1	1	1	9
Grobler et al. [35]	1	1	1	0	1	1	1	0	0	1	0	0	7
Harries et al. [53]	1	1	1	1	1	1	1	0	0	1	1	1	10
Harries et al. [51]	1	1	1	1	1	1	1	0	0	1	1	1	10
Howard et al. [69]	1	1	1	1	1	1	0	0	0	1	1	1	9
Jones et al. [59]	1	1	1	1	1	1	0	0	0	1	1	1	9
Kobal et al. [64]	1	1	1	1	1	1	0	0	0	1	1	1	9
Krause et al. [65]	1	1	1	1	1	1	1	0	0	1	1	1	10
Lombard et al. [57]	1	1	1	1	1	1	1	0	0	1	1	1	10
Nutton et al. [62]	1	1	1	1	1	1	0	0	0	1	1	1	9
Parsonage et al. [66]	1	1	1	1	1	1	0	0	0	1	1	1	9
Pienaar & Spamer [43]	1	1	1	0	1	1	1	0	0	1	1	0	8
Pienaar et al. [46]	1	1	1	1	1	1	1	0	0	1	1	0	9
Pienaar & Coetzee [48]	1	1	1	1	1	1	0	0	0	1	1	1	9
Plotz [63]	1	1	1	0	1	1	1	0	0	0	1	0	7
Sedeaud [104]	1	1	1	1	1	1	0	0	0	1	1	1	9
Smart & Gill [41]	1	1	1	1	1	1	0	0	0	1	1	1	9
Spamer [50]	1	1	1	0	1	0	0	0	0	1	0	0	5
Spamer & Hattingh [49]	1	0	1	0	1	1	1	0	0	1	1	0	7
Spamer & Winsley [44]	1	1	1	0	1	1	1	0	0	1	0	0	7
Spamer & Winsley [45]	1	1	1	0	1	1	1	0	0	1	0	0	7
Spamer & De la Port [33]	1	1	1	0	1	1	1	0	0	1	0	0	7
Spamer et al. [42]	1	1	1	0	1	1	1	0	0	1	1	0	8
Speirs et al. [61]	1	1	1	1	1	1	1	0	0	1	1	1	10
Van Gent & Spamer [34]	1	1	1	0	1	0	1	0	0	1	0	0	6
Vaz et al. [36]	1	1	1	1	1	1	1	0	0	1	1	1	10
Walsh et al. [52]	0	1	1	1	1	1	0	0	0	1	1	1	8
Weakley et al. [60]	1	1	1	1	1	1	1	0	0	1	1	1	10
Wood et al. [67]	1	1	1	1	1	1	1	0	0	1	1	1	10

1 = yes, 0 = no or unable to determine (where applicable)

### Data collection methods

The methods and outcome variables used to assess the physical qualities of age grade RU players are shown in Tables 4–7. Testing methods were placed into the following groups based on their purpose; body composition, strength, power, speed, change of direction, aerobic and anaerobic endurance.

**Table 4:** Body composition tests and outcome measures reported by studies.

Characteristic	Test	Output measure	Reference
Body composition	Bioelectrical impedance analysis	Body fat percentage	[51,52]
		Muscle mass	[53,56]
	DXA Scan	Body fat percentage	[54]
		Fat mass	
		Lean mass	
		Fat-free mass	
	Sum of 6 skinfolds	Body fat percentage	[47–50]
		Mm	[47,48]
		Fat free mass	[47]
	Sum of 7 skinfolds	Body fat percentage	[31–36]
		Mm	[30–38]
		Lean mass ratio	[30]
	Sum of 8 skinfolds	Body fat percentage	[41–46]
		Mm	[39–43]
		Fat free mass	[41]

*DXA* Dual-energy X-ray absorptiometry

**Table 5:** Strength and power tests and outcome measures reported by studies.

Characteristic	Test	Output measure	Reference
Strength	Back squat 1RM	1RM	[38]
		Relative 1RM	
	Back squat 3RM	Estimated 1RM	[30,61]
		3RM	[60]
		Relative 3RM	
	Back squat 5RM	Estimated 1RM	[51,53]
	Back squat (heavy)	Estimated 1RM	[36]
	Bench press 1RM	1RM	[31–33,38,57]
		Relative 1RM	[38]
	Bench press 1-4RM	Estimated 1RM	[58]
	Bench press 3RM	Estimated 1RM	[30]
		3RM	[40,59,60]
		Relative 3RM	[40,60]
	Bench press 6-10RM	Estimated 1RM	[41]
	Box squat 1-4RM	Estimated 1RM	[58]
	Box squat 6-10RM	Estimated 1RM	[41]
	Chin up 3RM	3RM	[40,59,60]
		Relative 3RM	[40,60]
	Chin up 6-10RM	Estimated 1RM	[41]
	Flexed arm hang	Max time	[34,36,44,50]
	Front squat 3RM	3RM	[40]
		Relative 3RM	
	Grip strength dynamometer	Maximum force (kg)	[35,36,46,62,63]
	Isometric mid-thigh pull	Peak force	[40]
		Peak rate of force development	
	Press up	Maximum reps	[36]
		Maximum reps in 60s	[31–33,35,37,38]
	Prone row	3RM	[40]
		Relative 3RM	
	Pull ups	Maximum reps	[2,36,46,57]
		Maximum reps in 60s	[33]
	Rear foot elevated split squat 3RM	Estimated 1RM	[61]
	Sit up	Maximum reps in 40s	[36]
		Maximum reps in 60s	[35]
	Split squat 3RM	3RM	[40]
		Relative 3RM	
	Wall sit	Time	[37,38]
Power	2kg medicine ball throw	Distance	[37,38]
	3kg medicine ball throw	Distance	[48]
	6s Watt Bike	Peak power output	[69]
	Bench throw (60% 1RM)	Peak power	[58]
	Countermovement jump	Height	[40,47,53,60,64–68]
		Peak power	[30,40,60]
		Force	[40,60]
	Countermovement jump (10kg)	Height	[53]
	Horizontal jump	Distance	[49]
	Squat jump	Height	[47,64]
	Squat jump (60% 1RM)	Peak power	[58]
	Triple hop	Distance	[67]
	Vertical jump	Height	[33,34,46,48–50,63,36–38,41–45]
		Power	[41,48]

*RM* Repetition maximum

**Table 6:** Speed and change of direction tests and outcome measures reported by studies.

Characteristic	Test	Output measure	Reference
Speed	0-5m	Velocity	[39,40]
		Acceleration	[39,40]
		Momentum	[39,40]
	0-10m	Velocity	[71]
		Momentum	[60,71]
	5m	Time	[39,40,48]
	5-10m	Velocity	[39,40]
		Acceleration	[39,40]
		Momentum	[39,40]
	8-12m	Velocity	[69]
		Momentum	[69]
	10m	Time	[31–37,39–42,48,53,57,59–61,64–68]
	10-20m	Velocity	[39,40]
		Acceleration	[39,40]
		Momentum	[39,40]
	15m	Time	[47,72]
	20m	Time	[36,37,64,66,38–41,48,53,59,60]
	20-40m	Velocity	[39,40]
		Acceleration	[39,40]
		Momentum	[39,40]
	30m	Time	[34,36,41,47,49,65]
	30-40m	Time	[65]
		Velocity	[71]
		Momentum	[60,71]
	35m	Time	[35]
	40m	Time	[31–33,36–40,57,59–61,64–66,70]
	50-yard (45.7m)	Time	[42–46,63]
	50m	Time	[36]
	60m	Time	[41]
Change of direction	505	Time	[40]
	Bloomfield agility test	Time	[49]
	Illinois	Time	[31–34,36]
	L-run	Time	[37,38]
	Pro agility	Time	[61,64]
	T-test	Time	[34,48]
	Zig-zag 15m	Time	[72]
	Zig-zag 30m	Time	[63]
	Zig-zag 45˚	Time	[64]

**Table 7:** Aerobic capacity and anaerobic endurance tests and outcome measures reported by studies.

Characteristic	Test	Output measure	Reference
Aerobic capacity	1500m	Time	[41]
	30-15IFT	Last completed stage	[39,40,73]
	MSFT	Completed stages	[32,57]
		Estimated VO_2_max	[30,35,36,47]
	YYE1	Distance	[64]
	YYIR1	Distance	[37,38,40,59,66,68,73]
Anaerobic endurance	400m	Time	[41]
	500m	Time	[46]
	150m shuttle test	Distance	[67]
	250m shuttle test	Distance	[35]
	Wingate anaerobic test	Peak power	[48]
		Average power	
		Total work	
		Fatigue rate	

*30-15IFT* 30–15 intermittent fitness test

*MSFT* Multistage shuttle run

*YYE1* Yo-Yo endurance test level 1

*YYIR1* Yo-Yo intermittent recovery test level 1

#### Body composition

Body composition was assessed in twenty-five of the forty-two studies (60%), with five testing methods used (Table 4). Skinfolds taken from 7 [30–38] and 8 [39–46] sites were the most commonly used procedures, performed in nine and seven studies respectively. Skinfolds at 6 sites [47–50], bioelectrical impedance analysis [51–53] and dual-energy X-ray absorptiometry (DXA) [54] were also used to assess body composition. Body fat percentage (*n* = 18) was the most frequently reported variable as it can be calculated from all testing methods [31–36,42–52,54,55]. The sum of skinfolds, a variable unique to skinfold testing, was reported fourteen times [30–40,42,43,47,48,55]. Other variables included the lean mass ratio [30] and the calculation of absolute measures of fat free mass [47,54,55], muscle mass [53,56], fat mass [54] and lean mass [54].

#### Muscular strength

Strength testing was performed in twenty-four studies (57%) (Table 5). Bench press was the most common strength test used in twelve studies [30–33, 37,38,40,55,57–60,56]. Both 1RM [31–33,38,57] and 3RM [30,40,59,60] bench press were more commonly used in comparison to 1-4RM [58] and 6-10RM [41]. Bilateral squat variations (back squat, box squat and front squat), were frequently used to measure lower body strength with the 3RM back squat most regularly performed [30,60,61] compared to a 1RM [38], 5RM [51,53], “heavy” loads [36], front squat 3RM [40], box squat 1–4 RM [58] or box squat 6-10RM [41]. Other external load exercises included chin up [40,41,59,60] and split squat variations [40,61]. The results were often reported as a 1RM [31–33,38,57] or an estimated 1RM [30,36,41,51,53,58,61] when a multiple RM protocol was employed. Three studies chose to report the raw 3RM [40,59,60] and relative strength values [38,40,60].

A variety of bodyweight exercises were included in the literature, with seven studies assessing upper body strength using press ups [31–33,35–38] and five using pull ups [32,33,36,46,57]. Abdominal strength was assessed using sit ups [35,36]. Maximum repetitions in a given time period was the most popular output measure with four studies using a 60s period [31–33,35,37,38] and one using a 40s period [36].

Although grip strength testing [35,36,45,62,63] and flexed arm hang [34,36,44,50] were regularly observed throughout the literature, isometric testing methods were the least common with only wall sits [37,38] and isometric mid-thigh pull (IMTP) [40] added to this group. Flexed arm hang and wall sits were reported for a maximum time, while the grip test and IMTP measured peak force. Peak rate of force development was also reported for the IMTP [40].

#### Muscular power

Twenty-nine studies (69%) directly (*n* = 7) or indirectly (*n* = 25) assessed power output within age grade RU players (Table 5). Jump variations were commonly used to assess lower body power with the vertical jump (VJ) [30,33,34,36–38,41–46,48–50,63] and countermovement jump (CMJ) [40,47,53,60,64–68] being most popular. Although similar, the VJ makes use of an arm swing during the movement and is therefore classified as a different test. For both the VJ and CMJ, jump height (*n* = 15 and *n* = 9, respectively) and peak power (*n* = 2 and *n* = 3, respectively) were recorded (Table 5). Peak force was also used to quantify performance for the CMJ [40,60]. Other vertical jump variations used included a squat jump [47,64], 10 kg squat jump [53] and 60% 1RM squat jump [58]. Further tests of lower body power used in a single study included jumps in a horizontal vector (horizontal jump and triple hop) and 6s Watt bike peak power output [49,67,69]. Only two studies carried out tests of upper body power reporting peak power for bench throw (60% 1RM) [58] and distance for a 3 kg medicine ball throw [48].

#### Linear speed

Sprinting speed was the most reported physical quality shown in Table 6. Thirty-two of the forty-two (76%) studies reported speed related qualities. Time was the most common variable recorded over a range of distances from 5m to 60m (Table 4). Of the ten distances reported, 10m [31–37,39–42,48,53,57,59–61,64–68], 20m [36–41,48,53,59,60,64,66] and 40m [31–33,36–40,57,59–61,64–66,70] were observed in ten or more articles. In addition to time, average velocity, in acceleration and momentum were also reported [39,40,60,69,71].

#### Change of direction

There were fourteen studies (33%) that evaluated the change of direction performance of age grade RU players (Table 6). Of the seven tests used, only four change of direction tests were performed in multiple studies; Illinois agility test [31–34,36], Pro agility test [61,64], T-test [34,48] and L-run [37,38]. In addition to this the 505 [40] and three variations of the zig-zag test, 15 m [72], 30 m [63] and 45˚ [64], were all used in single studies.

#### Aerobic capacity

Table 3 shows the sixteen (38%) studies that investigated the aerobic capacity of age grade RU players. The MSFT was reported as both number of stages completed [32,57] and estimated VO_2_max [30,35,36,47]. Additionally, other aerobic tests that were continuous in nature included the YYE1 [64] and 1500m run [41]. The YYIR1 [37,38,40,59,66,68,73] and 30-15IFT [39,40,73] provided an intermittent assessment of aerobic capacity reported as total distance covered and final running velocity, respectively.

#### Anaerobic endurance

Only five studies (12%) tested the anaerobic endurance capabilities of age grade RU players (Table 3). Two tests reported the fastest time to cover 400 m [41] and 500 m [46], while two tests reported the distance covered during the 150 m [67] and 250 m [35] shuttle tests. Additionally, the Wingate anaerobic test was also used to assess anaerobic power through peak power, average power, total work and fatigue rate [48].

### Physical qualities of age grade rugby union players

A total of thirty-one papers were selected to present and compare the physical qualities of age grade RU players by age grade and position. The analysis included data for the following physical qualities: anthropometrics (height, body mass and body fate percentage), strength (bench press and squat variations), power (VJ height, CMJ height and peak power), speed (10, 20, 30 and 40 m), change of direction (Illinois agility test, T-test, pro agility, L-run and 505) and aerobic capacity tests (MSFT, YYE1, YYIR1and 30-15IFT). The age of players ranged from U11 to U20 years. Throughout this review, positions have either been grouped as all, separated into units (backs and forwards), or divided into four (back-line, half backs, loose forwards and tight forwards) or nine (full back, wing, centre, fly half, scrum half, loose forward, lock, hooker and prop) positional groups, as per the published paper.

#### Anthropometric qualities

Height and body mass were reported in all thirty-one articles identified for the second aim. Height and body mass for U11 to U20 are reported in Table 8. The youngest age group (U11) were observed to be both the shortest and lightest (146.6 ± 5.8 cm and 36.4 ± 5.6 kg), whereas the tallest population competed at U18 (185.6 ± 6.6 cm) and heaviest at U20 (99.0 ± 13.0 kg) [45,46,57]. Differences were observed in heights reported for U11 (146.6 cm), U12 (147.3–155.0 cm), U13 (163.0 cm), U14 (170.0–172.0 cm), U15 (169.7–175.0 cm) and U16 (168.0–182.8 cm) [30,33,35,37,40,42,44,46,47,62,64,66,73]. Following U16, differences become less clear with similar heights reported until U20 (U17; 177.2–180.0 cm, U18; 178.0–185.6 cm, U19; 172.0–177.0 cm, U20; 184.0 cm) [30,31,33,38,40,45,57,59,60,62–64,71,73,74]. At all age grades (U13, U15, U16, U18, U19 and U20), forwards are shown to be taller than backs. Durant [30] reported locks to be the tallest for U16 (187.2 ± 5.5 cm) and U18 (194.2 ± 5.2 cm), with scrum halves the smallest (165.9 ± 10.3 cm & 167.8 ± 5.6 cm).

**Table 8:** Anthropometrics and body composition of age grade rugby union players.

Age group	Playing position	Playing level	Height (cm)	Body mass (kg)	Body fat (%)
U11	All [46]	Club	146.6 ± 5.82	36.4 ± 5.56	15.30 ± 6.30 ^a^
U12	All [44]	School	147.27 ± 6.24	42.55 ± 5.65	20.28 ± 4.90 ^a^
	All [44]	School	150.88 ± 7.39	42.20 ± 6.57	19.23 ± 5.89 ^a^
	All [62]	School	155.0 ± 7.6	48.0 ± 9.2	
U13	All [62]	School	163.0 ± 7.8	54.0 ± 10.8	
	Back-line [34]	Provincial	170.00	56.33	12.76 ^b^
	Half Back [34]	Provincial	160.50	48.00	14.32 ^b^
	Loose Forward [34]	Provincial	170.00	60.00	12.59 ^b^
	Tight Forward [34]	Provincial	173.13	66.19	17.75 ^b^
U14	All [35]	School	172.0 ± 6.0	67.45 ± 13.23	13.87 ± 8.06 ^b^
	All [62]	School	170.0 ± 7.5	61.0 ± 9.8	
U15	All [35]	School	175.0 ± 6.0	75.90 ± 13.26	15.55 ± 8.13 ^b^
	All [62]	School	175.0 ± 7.0	68.0 ± 11.4	
	All [64]	Club	169.7 ± 12.1	63.8 ± 10.9	
	Backs [104]	Club	169.5 ± 6.5	60.8 ± 8.2	
	Forwards [104]	Club	175.9 ± 7.0	72.5 ± 9.8	
	Backs [49]	School	171.88 ± 6.17	63.5 ± 12.49	14.53 ± 3.41 ^c^
	Forwards [49]	School	176.60 ± 8.59	83.80 ± 13.10	21.52 ± 8.13 ^c^
U16	All [35]	School	180 ± 8	89.91 ± 17.09	18.86 ± 8.69 ^b^
	All [62]	School	179.0 ± 7.5	72.0 ± 10.2	
	All [37]	School	168 ± 8	61.2 ± 15.5	
	All [37]	School	167 ± 8	63.7 ± 9.09	
	All [42]	Provincial	179.71 ± 5.83	81.26 ± 8.31	13.66 ± 4.77 ^b^
	All [66]	Academy	176 ± 7	74 ± 14	
	All [40]	Academy	178.8 ± 7.1	79.4 ± 12.8	
	All [73]	Academy	177.2 ± 7.2	76.2 ± 13.1	
	All [47]	National draft camp	182.8 ± 5.1	86.9 ± 13.2	17.2 ± 6.8 ^c^
	All [33]	National	178.17 ± 7.57	79.50 ± 13.63	15.04 ± 4.18 ^b^
	All [31]	National	178.17 ± 7.57	79.50 ± 13.63	15.04 ± 4.18 ^b^
	Backs [39]	Academy	175.6 ± 6.6	70.5 ± 10.8	
	Forwards [39]	Academy	181.9 ± 6.3	87.6 ± 8.1	
	Back-line [34]	Provincial	178.50	72.25	14.13 ^b^
	Half Back [34]	Provincial	172.50	68.00	12.17 ^b^
	Loose Forward [34]	Provincial	183.75	77.50	16.67 ^b^
	Tight Forward [34]	Provincial	183.88	82.75	18.39 ^b^
	All [32]	Provincial	175.6 ± 5.7	76.5 ± 8.2	14.5 ± 3.4 ^b^
	Full back [32]	Provincial	178.1 ± 5.5	75.2 ± 6.8	13.0 ± 4.6 ^b^
	Wing [32]	Provincial	171.7 ± 5.2	68.4 ± 6.7	13.3 ± 2.9 ^b^
	Centre [32]	Provincial	173.4 ± 6.5	71.9 ± 9.1	12.0 ± 1.8 ^b^
	Fly half [32]	Provincial	173.0 ± 5.3	69.6 ± 5.3	13.6 ± 2.0 ^b^
	Scrum half [32]	Provincial	165.9 ± 10.3	60.8 ± 8.9	13.2 ± 4.2 ^b^
	Loose forward [32]	Provincial	180.8 ± 4.3	80.5 ± 7.3	14.2 ± 3.1 ^b^
	Lock [32]	Provincial	187.2 ± 5.5	87.1 ± 8.8	14.8 ± 4.6 ^b^
	Hooker [32]	Provincial	173.4 ± 3.1	79.5 ± 6.4	16.4 ± 2.4 ^b^
	Prop [32]	Provincial	177.5 ± 6.0	95.5 ± 14.1	20.0 ± 5.5 ^b^
U17	All [62]	School	180 ± 6.2	76 ± 12.8	
	All [64]	Club	177.2 ± 8.7	76.3 ± 13.1	
U18	All [60]	School	178 ± 7	80.1 ± 10.5	
	All [59]	School	179.2 ± 10.0	78.4 ± 12.9	
	All [45]	School	181.9 ± 7.4	87.8 ± 11.5	22.1 ± 6.8 ^a^
	All [62]	School	182.0 ± 8.1	84 ± 14.9	
	All [63]	School	181.86 ± 7.40	87.84 ± 11.52	
	All [45]	Provincial	185.6 ± 6.6	87.4 ± 14.3	15.8 ± 5.5 ^a^
	All [74]	Provincial	181.6 ± 8.3	88.5 ± 13.6	
	All [40]	Academy	183.5 ± 7.2	88.3 ± 11.9	
	All [73]	Academy	183.8 ± 7.1	88.4 ± 10.8	
	All [59]	Academy	184.0 ± 7.5	88.8 ± 12.2	
	All [31]	National	180.43 ± 9.04	86.83 ± 13.86	14.65 ± 4.06 ^b^
	All [33]	National	180.43 ± 9.04	86.83 ± 13.86	14.65 ± 4.06 ^b^
	Backs [52]	School	179.8 ± 5.6	75.9 ± 8.0	11.3 ± 2.8 ^d^
	Forwards [52]	School	182.5 ± 6.5	85.5 ± 10.8	14.7 ± 4.6 ^d^
	Backs [68]	Academy	176 ± 7	80 ± 12	
	Forwards [68]	Academy	188 ± 7	100 ± 6	
	Backs [39]	Academy	178.9 ± 3.9	78.7 ± 6.9	
	Forwards [39]	Academy	188.1 ± 6.2	93.8 ± 7.0	
	Back-line [34]	Provincial	182.75	77.50	14.03 ^b^
	Half Back [34]	Provincial	172.0	68.67	15.30 ^b^
	Loose Forward [34]	Provincial	188.0	83.50	16.69 ^b^
	Tight Forward [34]	Provincial	187.86	96.57	23.58 ^b^
	Back [54]	School	178 ± 5.63	73.65 ± 6.61	14.34 ± 3.08 ^e^
	Forward [54]	School	182 ± 7.11	83.63 ± 10.53	18.46 ± 5.91 ^e^
	Back three [54]	School	178	72.46	12.71 ^e^
	Centre [54]	School	181	78.18	14.90 ^e^
	Out half [54]	School	179	75.48	17.83 ^e^
	Scrum half [54]	School	175	69.40	14.38 ^e^
	Back row [54]	School	179	78.06	15.01 ^e^
	Second row [54]	School	189	84.9	17.03 ^e^
	Hooker [54]	School	176	81.22	19.73 ^e^
	Prop [54]	School	181	92.45	24.46 ^e^
	All [32]	Provincial	179.2 ± 6.7	84.9 ± 8.3	14.3 ± 2.7
	Full back [32]	Provincial	177.6 ± 9.0	78.8 ± 6.6	12.1 ± 3.4 ^b^
	Wing [32]	Provincial	176.4 ± 8.2	77.7 ± 12.2	13.1 ± 1.6 ^b^
	Centre [32]	Provincial	179.1 ± 8.5	85.1 ± 9.9	13.8 ± 2.9 ^b^
	Fly half [32]	Provincial	177.6 ± 7.6	75.0 ± 8.2	13.3 ± 2.4 ^b^
	Scrum half [32]	Provincial	167.8 ± 5.6	70.3 ± 4.9	12.9 ± 2.7 ^b^
	Loose forward [32]	Provincial	181.3 ± 6.3	88.2 ± 5.5	13.9 ± 1.8 ^b^
	Lock [32]	Provincial	194.2 ± 5.2	95.2 ± 8.4	14.2 ± 2.0 ^b^
	Hooker [32]	Provincial	178.8 ± 6.3	93.1 ± 5.7	15.3 ± 3.7 ^b^
	Prop [32]	Provincial	180.3 ± 3.8	100.8 ± 13.1	20.0 ± 4.2 ^b^
U19	All [38]	School	172 ± 8	75.9 ± 11.6	
	All [38]	School	173 ± 6	77.5 ± 9.58	
	All [64]	Club	177.0 ± 7.1	82.5 ± 18.2	
	Backs [49]	Club	176.79 ± 8.53	76.65 ± 8.53	10.12 ± 2.86 ^c^
	Forwards [49]	Club	184.04 ± 5.98	96.22 ± 10.90	15.46 ± 4.76 ^c^
	Backs [68]	Academy	179 ± 6	83 ± 12	
	Forwards [68]	Academy	190 ± 8	102 ± 7	
	Backs [36]	National	177.4 ± 3.4	78.2 ± 6.9	
	Forwards [36]	National	180.8 ± 4.7	90.3 ± 18.7	
	Back-line [34]	Provincial	183.0	82.80	14.75 ^b^
	Half Back [34]	Provincial	179.33	77.00	16.48 ^b^
	Loose Forward [34]	Provincial	184.25	86.38	16.97 ^b^
	Tight Forward [34]	Provincial	185.86	101.14	25.93 ^b^
U20	All [30]	University		90.7 ± 12.5	
	Backs [30]	University		82.4 ± 8.3	
	Forwards [30]	University		98.0 ± 11.1	
	Backs [68]	Academy	178 ± 7	84 ± 9	
	Forwards [68]	Academy	190 ± 8	105 ± 5	
	All [71]	National	184 ± 10	93.2 ± 12.3	
	Backs [71]	National		83.7 ± 7.8	
	Forwards [71]	National		101.0 ± 9.6	
	All [57]	National	184 ± 7	99 ± 13	
	Backs [57]	National	178.7 [175.4, 181.7]	87.7 [83.5, 91.9]	
	Forwards [57]	National	187.2 [184.3, 190.1]	107.4 [103.0, 110.6]	
	Backs [49]	Club	177.73 ± 6.25	78.84 ± 8.64	10.58 ± 2.47 ^c^
	Forwards [49]	Club	182.86 ± 6.75	96.05 ± 11.57	14.63 ± 5.27 ^c^

Data expressed as mean ± SD or mean [95% confidence interval]

^a^ Sum of 8 skinfolds

^b^ Sum of 7 skinfolds

^c^ Sum of 6 skinfolds

^d^ Bioelectrical impedance analysis

^e^ DXA scan

Differences in body mass were observed from U11 to U16 (U11; 36.4 kg, U12; 42.2–48 kg, U13; 54.0 kg, U14; 67.5 kg, U15; 63.8–75.9 kg & U16; 61.2–89.9 kg) [31,33,35,37,40,42,44,46,47,62,64,66,73]. Similar results are reported between U16 to U19 (U17; 76.0–76.3 kg, U18; 78.4–87.8 kg & U19; 75.9–82.5 kg) [31–33,38,40,45,57,59,60,62–64,73]. At all age groups forwards were heavier than backs, with tight forward and specifically prop the heaviest at U13, U15, U16, U18, U19 and U20.

#### Body composition

Body composition was presented as a body fat percentage in sixteen studies (Table 8). The highest body fat was recorded at U20 (22.1 ± 6.8%) [45]. All other age grades are reported to have a mean body fat percentage <16%, with the exception U12’s [44] and one study at U16 [47]. The lowest reported body fat was observed in U16 provincial players (13.66 ± 4.77%) [42]. Apart from loose forwards at U13, all backs had a lower body fat percentage compare to forwards [32,34,49,52].

#### Muscular strength

Nine papers from those identified reported the use of bench press for U16, U18, U19 and U20 RU players (Table 9). Six papers reported bench press performance using 1RM or estimated 1RM for U16 (77.1–82.9 kg), U18 (95.3–105.9 kg), U19 (80.6–90.5 kg) and U20 (108.1–135 kg) [30–33,38,57]. Positional differences were identified at U16, U18 and U20 (Table 9). Lombard et al. [57] and Ball et al. [30] found U20 estimated 1RM bench press to be greater in forwards (114.9–137.9 kg) than backs (100.4–129.6 kg). Scrum half had the lowest upper body strength at both U16 and U18 (63.0 ± 6.7 kg and 81.9 ± 13.1 kg, respectively) [32]. At U16 props achieved the greatest 1RM (97.5 ± 16.9 kg), while hookers the greatest at U18 (107.0 ± 4.5 kg) [32].

**Table 9:** Bench press and squat variation results for age grade rugby union players.

Age group	Position	Playing level	Bench Press (kg)	Squat Variation (kg)
U16	All [31]	National	82.89 ± 15.87 ^b^	
	All [33]	National	82.89 ± 15.87 ^b^	
	All [32]	Provincial	77.1 ± 11.8 ^b^	
	Full back [32]	Provincial	72.5 ± 6.1 ^b^	
	Wing [32]	Provincial	69.6 ± 7.8 ^b^	
	Centre [32]	Provincial	72.2 ± 18.4 ^b^	
	Fly half [32]	Provincial	73.0 ± 9.7 ^b^	
	Scrum half [32]	Provincial	63.0 ± 6.7 ^b^	
	Loose forward [32]	Provincial	82.7 ± 18.4 ^b^	
	Lock [32]	Provincial	80.6 ± 12.1 ^b^	
	Hooker [32]	Provincial	83.0 ± 10.4 ^b^	
	Prop [32]	Provincial	97.5 ± 16.9 ^b^	
U18	All [60]	School	68.5 ± 12.8 ^a^	77.4 ± 32.6 ^d^
	All [59]	School	67.7 ± 15.5 ^a^	
	All [59]	Academy	88.3 ± 12.7 ^a^	
	All [40]	Academy	82.6 ± 10.8 ^a^	88.6 ± 10.8 ^e^
	All [31]	National	105.94 ± 21.38 ^b^	
	All [33]	National	105.94 ± 21.38 ^b^	
	All [32]	Provincial	95.3 ± 16.7 ^b^	
	Full back [32]	Provincial	95.0 ± 13.2 ^b^	
	Wing [32]	Provincial	94.4 ± 23.2 ^b^	
	Centre [32]	Provincial	98.2 ± 12.1 ^b^	
	Fly half [32]	Provincial	82.1 ± 20.8 ^b^	
	Scrum half [32]	Provincial	81.9 ± 13.1 ^b^	
	Loose forward [32]	Provincial	101.4 ± 21.3 ^b^	
	Lock [32]	Provincial	95.0 ± 15.8 ^b^	
	Hooker [32]	Provincial	107.0 ± 4.5 ^b^	
	Prop [32]	Provincial	102.7 ± 26.3 ^b^	
U19	All [38]	School	80.6 ± 15.9 ^b^	90.5 ± 16.4 ^f^
	All [38]	School	90.5 ± 16.4 ^b^	98.4 ± 14.8 ^f^
	Backs [36]	National		130.25 ± 30.07 ^g^
	Forwards [36]	National		151.32 ± 23.66 ^g^
U20	All [30]	University	108.1 ± 17.0 ^c^	139.5 ± 24.0 ^h^
	Backs [30]	University	100.4 ± 17.0 ^c^	130.0 ± 35.2 ^h^
	Forwards [30]	University	114.9 ± 14.1 ^c^	147.6 ± 21.6 ^h^
	All [57]	National	135 ± 22 ^b^	
	Backs [57]	National	129.6 [118.7, 141.3] ^b^	
	Forwards [57]	National	137.9 [127.1, 147.1] ^b^	

Data expressed as mean ± SD or mean [95% confidence interval]

^a^ 3RM bench press

^b^ 1RM bench press

^c^ 1RM bench press estimated from 3RM

^d^ 3RM back squat

^e^ 3RM front squat

^f^ 1RM back squat

^g^ 1RM back squat estimated from heavy weight

^h^ 1RM back squat estimated from 3RM

Back squat variations were used in five articles to quantify lower body strength at U18, U19 and U20 (Table 9). Academy U18 players are reported to have a greater 3RM front squat (88.6 ± 10.8 kg) compared to their school peers 3RM back squat (77.4 ± 32.6 kg) [40,60]. U20 predicted 1RM back squat was greater than U19 1RM back squat (139.5 and 90.5–98.4 kg, respectively) [30,38].Forwards were observed to have a greater 1RM compared to backs for U19 national (130.25 ± 30.07 vs 151.32 ± 23.66 kg) and U20 university players (130.0 ± 35.2 vs 147.6 ± 21.6 kg) [30,36].

#### Muscular power

**Fig 2:**
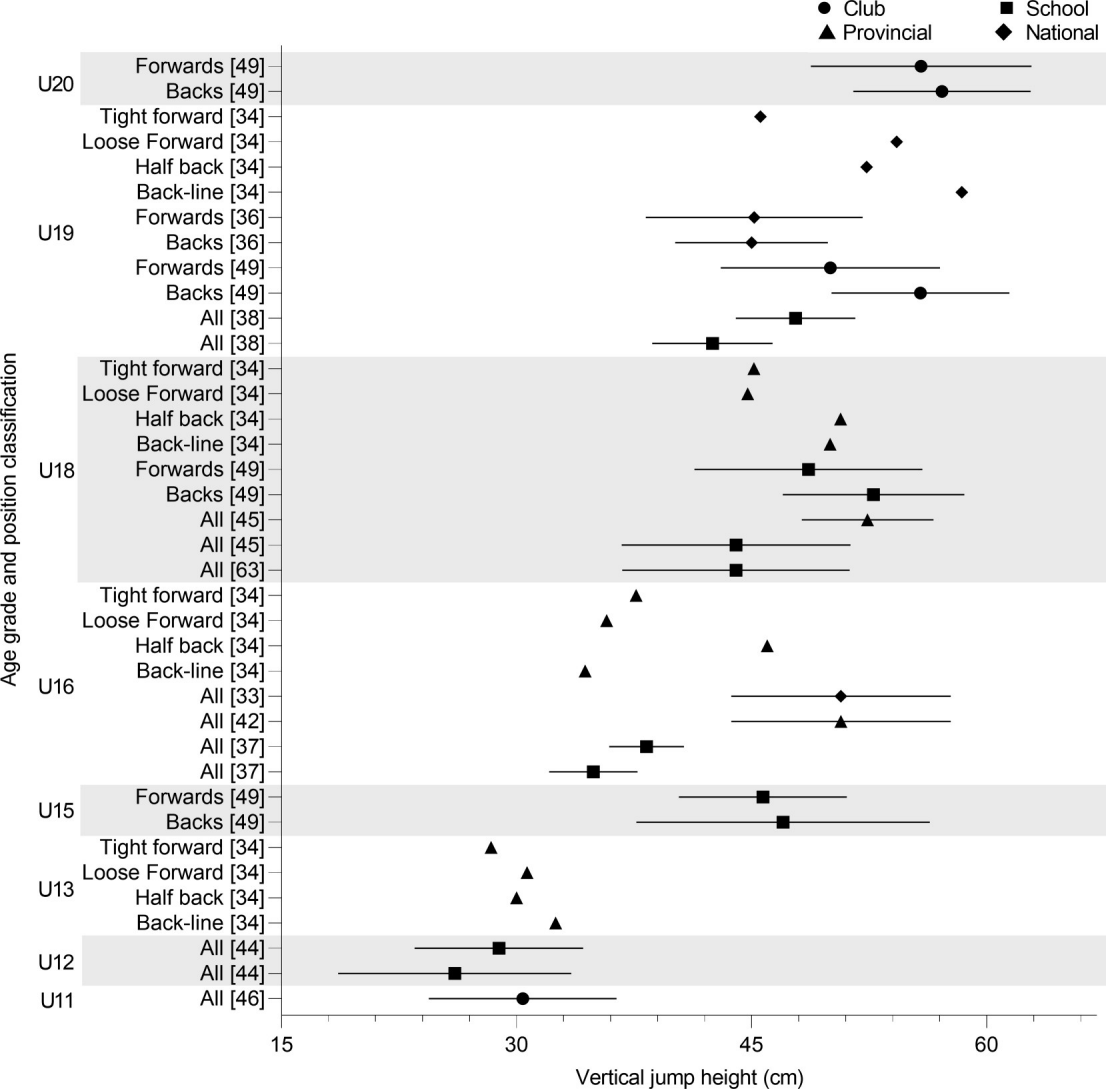
Vertical jump height of age grade rugby union players; mean and standard deviation as reported.

VJ performance for age grades were reported in seventeen studies (Fig 2). The lowest reported VJ performance for a whole team was at U12 (26.1–28.9 cm) [44]. This was lower than the value reported for younger U11 players (30.4 ± 6.0 cm) [46]. With the exception of the U16 results reported by Chiwaridzo et al. [37] (34.9–38.3 cm), similar VJ height means are reported for U16 (50.7 cm), U18 (44.0–52.4 cm) and U19 (42.5–47.8 cm) [33,38,42,45,63]. The greatest VJ performance was observed in U18 regional players (52.4 ± 4.2 cm) [45].

Spamer & Hattingh [49] reported backs to have a higher VJ height than forwards at U15 (47.0 ± 9.4 vs 45.7 ± 5.4 cm), U18 (52.8 ± 5.8 vs 48.6 ± 7.3 cm), U19 (50.7 ± 5.8 vs 50.0 ± 7.0 cm) and U20 (57.1 ± 5.7 vs 55.8 ± 5.7 cm). Another study found there to be no difference between backs and forwards at the U19 level (45.0 ± 4.9 cm vs 45.2 ± 6.9 cm) [36]. Further positional breakdown showed backline players have the highest VJ at U13 (32.5 cm) and U19 (58.4 cm), but the lowest at U16 (34.5 cm) [34]. Tight forwards produced the lowest VJ heights during the VJ test at U13 (28.4 cm) and U19 (45.6 cm) [34].

**Fig 3:**
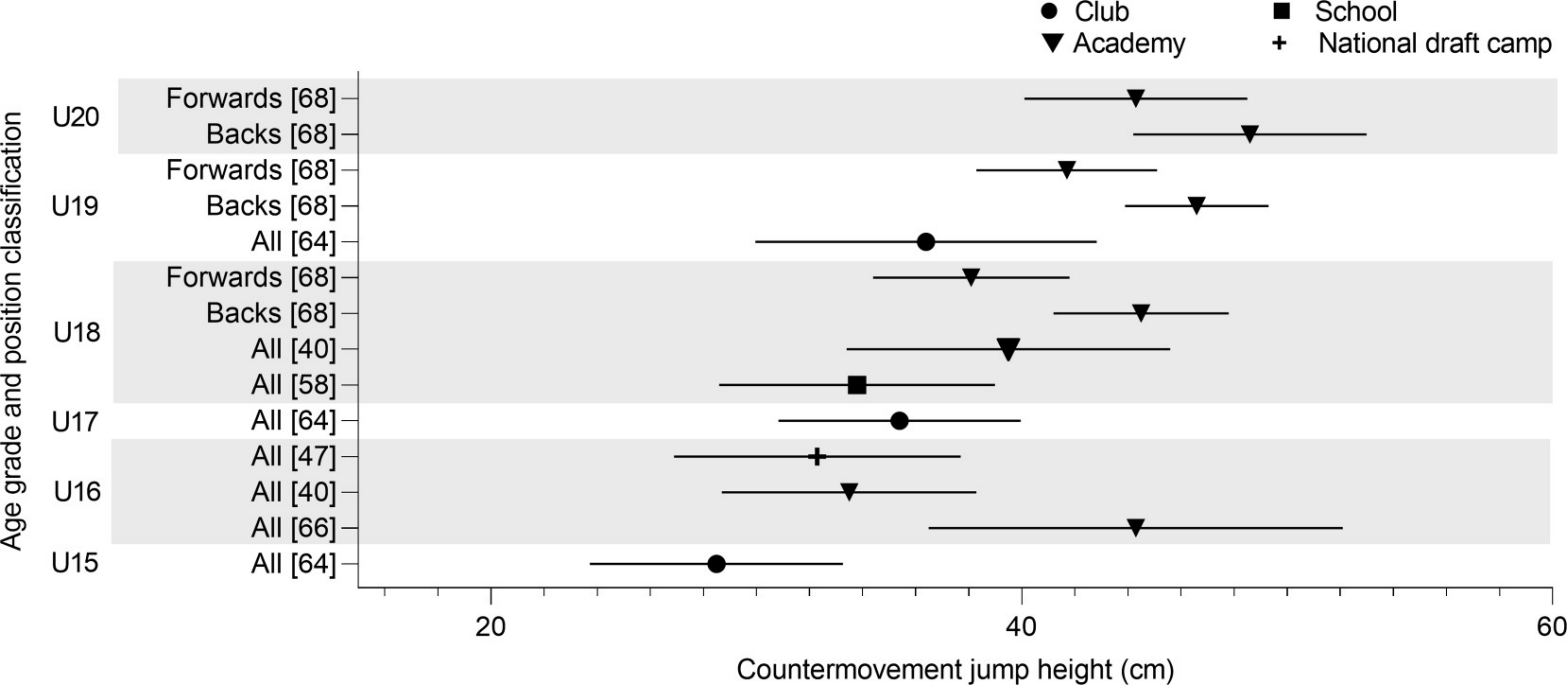
Countermovement jump height of age grade rugby union players; mean and standard deviation as reported.

CMJ height was reported for U15, U16, U17, U18, U19 and U20, by six articles (Fig 3). U15’s have the lowest CMJ heights (28.5 ± 4.8 cm) [64]. Similar team mean heights were reported from U16 to U19’s [40,47,60,64] with the exception of the U16 results reported by Parsonage et al. [66] (44.3 ± 7.8 cm). Backs were observed to jump higher than forwards for U18 (44.5 ± 3.3 vs 38.1 ± 3.7 cm), U19 (46.6 ± 2.7 vs 41.7 ± 3.4 cm) and U20 (48.6 ± 4.4 vs 44.3 ± 4.2 cm) [68].

**Fig 4:**
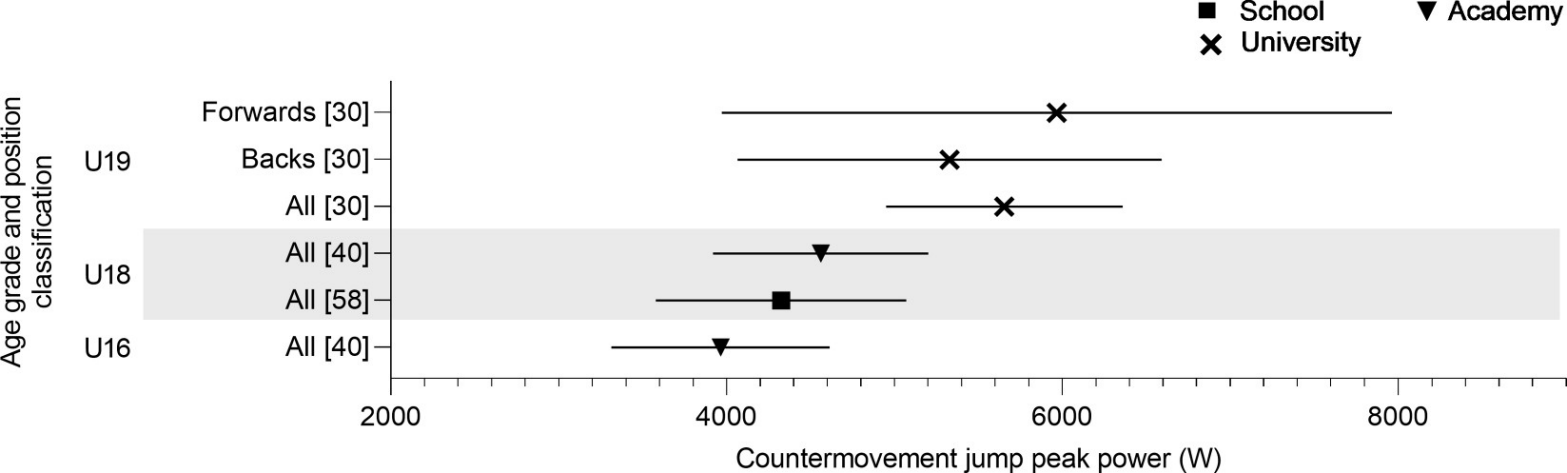
Countermovement jump peak power of age grade rugby union players; mean and standard deviation as reported.

Fig 4 shows the CMJ peak power was reported in three studies. Three age groups were assessed (U16, U18 and U20) with U16’s (3965 ± 650 W) producing the lowest peak power [40]. As age increased peak power also increased (U18; 4325–4561 W and U20; 5655 W) [30,40,60]. The only study that identified positional differences reported a greater peak power for forwards (5967 ± 1263 W) compared to backs (5328 ± 1263 W) for U20 players [30].

#### Linear speed

**Fig 5:**
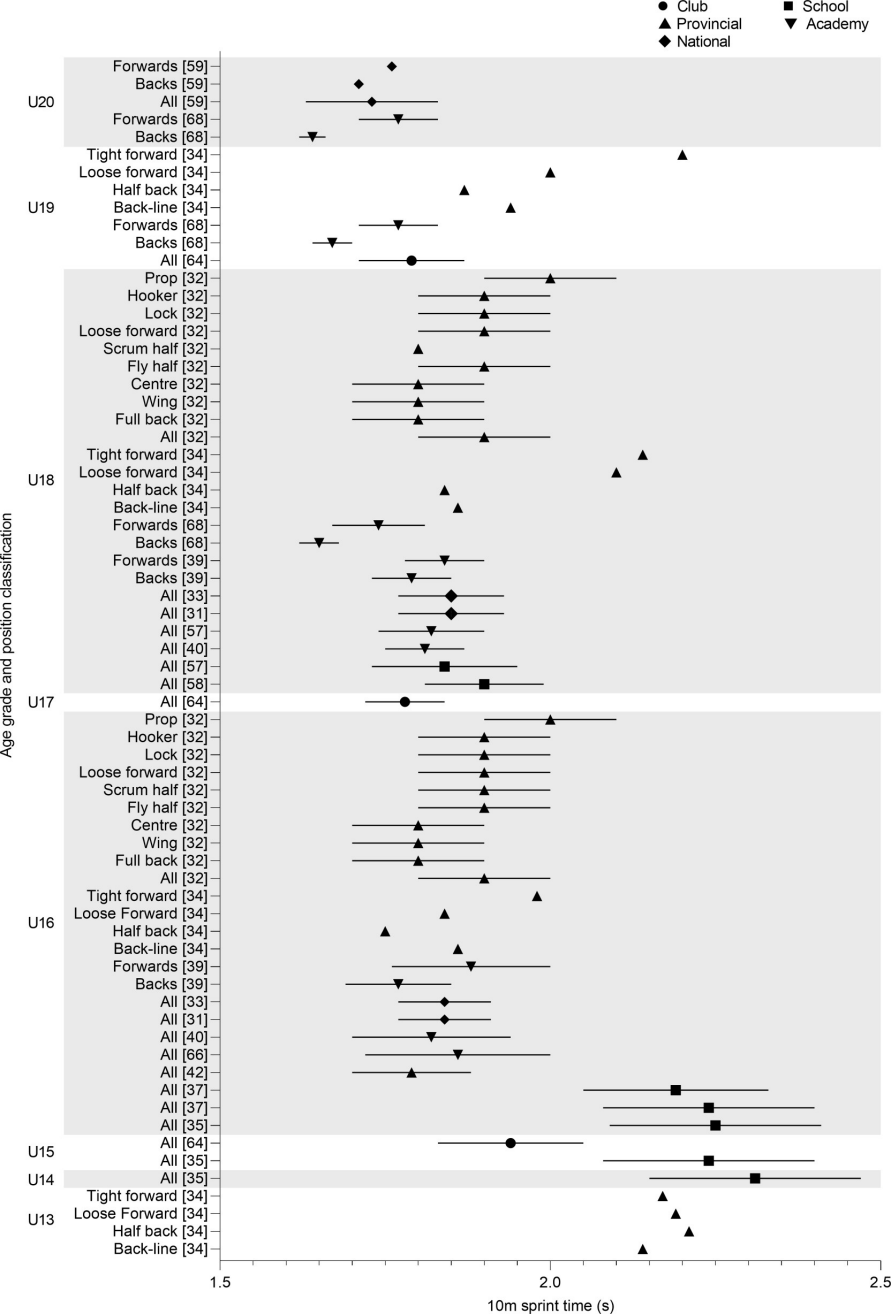
10m sprint time of age grade rugby union players; mean and standard deviation as reported.

Linear speed was reported from U13 to U20 by twenty articles over 10, 20, 30, and 40 m. Over the shortest distance (10 m), U14 school players (2.31 ± 0.16 s) were the slowest and U20 national players (1.73 ± 0.10 s) the fastest [35,57]. Differences in 10 m times were observed between U14 (2.31 s) and U16 (1.79–2.25 s), but 10m times remained similar from U16 to U20 (Fig 5). Positional comparisons found backs to be faster than forwards at U16, U18, U19 and U20 [39,57,68] except for U13 half backs who recorded 2.21 s compared to loose forwards 2.19 s and tight forwards 2.17 s [34]. Durandt et al. [32] observed all positions, except props, to run 10 m in under 1.90 s at both U16 and U18.

**Fig 6:**
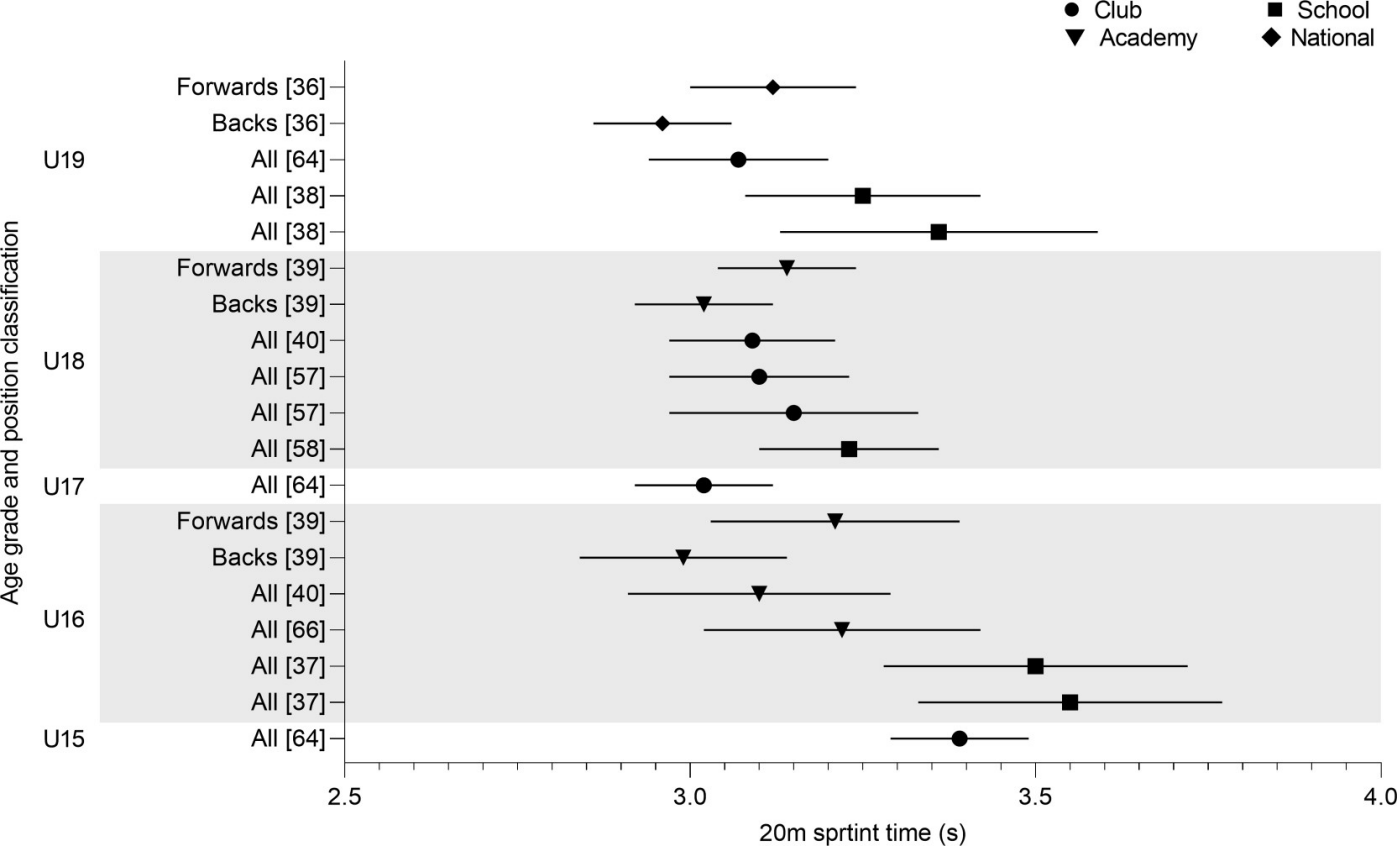
20m sprint time of age grade rugby union players; mean and standard deviation as reported.

20m sprint times are shown in Fig 6. The slowest time was observed in U16 school players (3.55 ± 0.22 s) [37], which was slower than U15 club level players (3.39 ± 0.10 s) [64]. With the exception of the times reported by Ciwaridzo et al. [37] faster times were observed in U16 (3.10–3.22 s) compared to U15 [40,66]. The U17 time (3.02 ± 0.10 s) reported by Kobal et al. [64] was the fastest team mean recorded, even compared to U18 (3.09–3.23 s) or U19 (3.07 ± 0.25 s) [40,59,60]. Differences between units showed backs to be faster than forwards at U16, U18, U19 and U20 [36,39].

**Fig 7:**
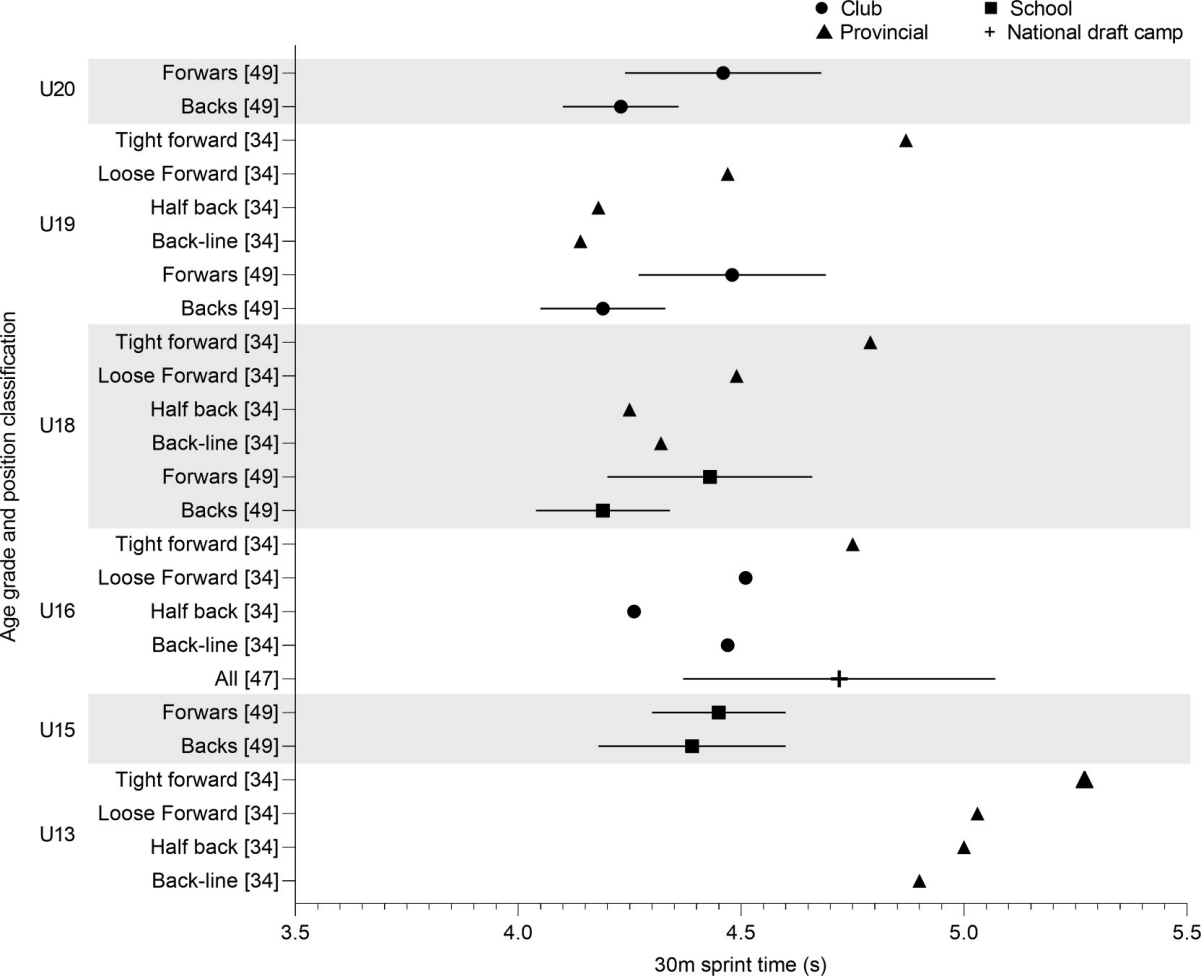
30m sprint time of age grade rugby union players; mean and standard deviation as reported.

Only one article reported the team mean for 30m sprint performance at U16 level (Fig 7) [47]. Spamer & Hattingh [49] found backs times increase from U15 (4.39 ± 0.21 s) to U18 (4.19 ± 0.15 s) and U19 (4.19 ± 0.14 s) before increasing at U20 (4.23 ± 0.13 s). The times observed for forwards are similar between age groups (U15; 4.45 ± 0.15 s, U18; 4.34 ± 0.23 s, U19; 4.48 ± 0.21 s, U20; 4.46 ± 0.22 s) but slower in comparison to backs [49].

**Fig 8:**
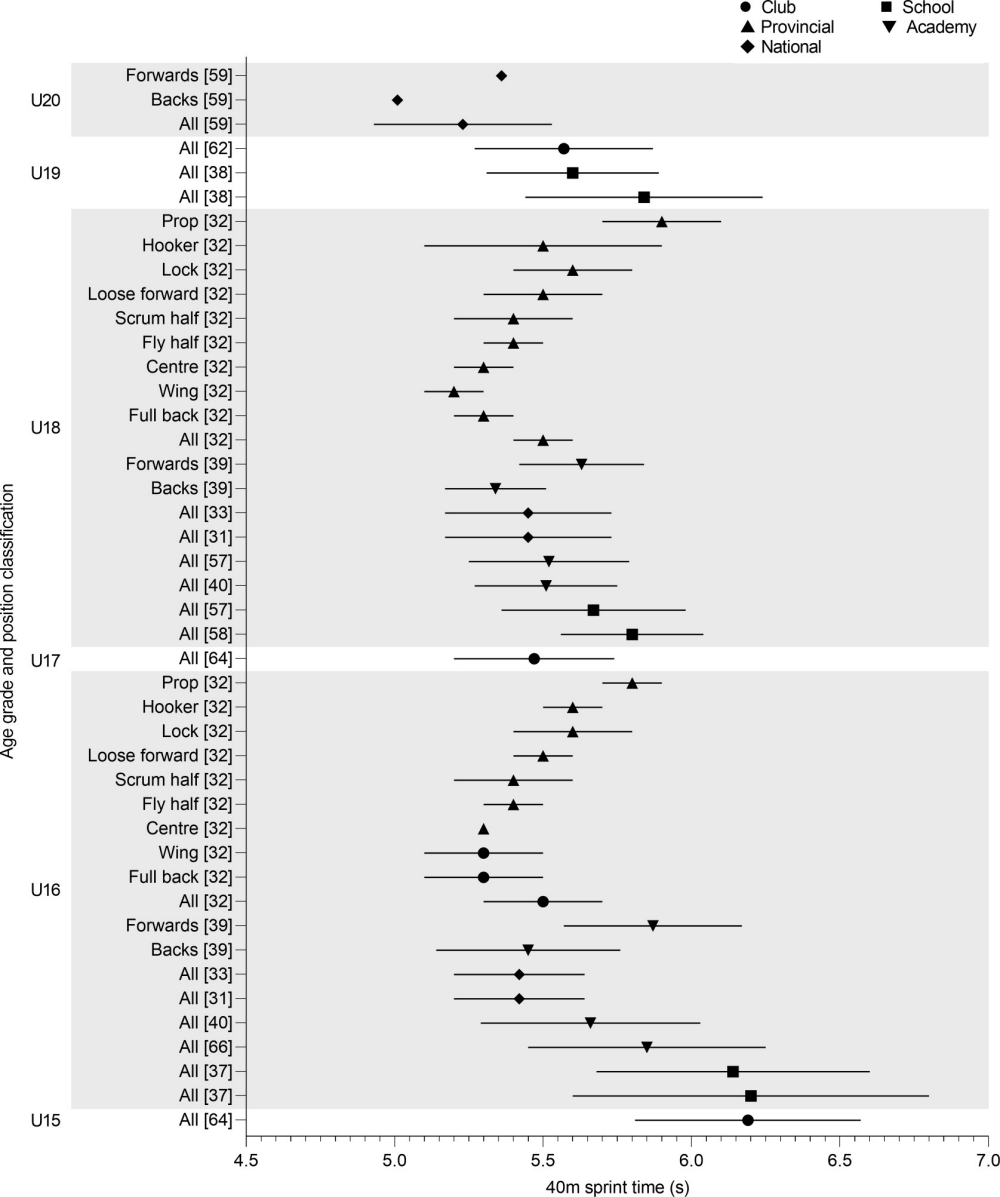
40m sprint time of age grade rugby union players; mean and standard deviation as reported.

Over 40m the slowest reported time was observed in U16 school players (6.20 ± 0.60 s) (Fig 8) [37]. Excluding school U16 school RU players [37], similar results were observed over 40m for U16 (5.42–5.85 s), U18 (5.45–5.80 s) and U19 (5.57–5.84 s) [31–33,38,40,66]. U20 national players recorded the fastest time (5.23 ± 0.30 s) [57]. Positionally, backs performed better than forwards at U16 (5.45 ± 0.31 s vs 5.87 ± 0.30 s), U18 (5.34 ± 0.17 s vs 5.63 ± 0.21 s) and U20 (5.01 s vs 5.36 s) [39,57]. Props cover 40m in the slowest time at U18 (5.90 ± 0.20 s) and U16 (5.80 ± 0.10 s), with all other positions producing times of 5.60 s or lower [32].

#### Change of direction

Eight studies have reported values for change of direction performance for age groups spanning U13 to U19 (Table 10). Using the Illinois agility test, Van Gent & Spamer [34] reported decreases in time from U13 (~19.20 s) to U19 (~16.28 s). Three other articles observed similar performance on the Illinois agility test between U16 (15.20–15.43 s) and U18 (15.00–15.39 s) players [31–33]. Kobal et al. [64] performed the pro-agility test to assess change of direction ability and found U15’s (5.34 ± 0.20 s) to be the slowest. Faster times were observed in the same study by U17’s (5.08 ± 0.18 s) and U19’s (5.02 ± 0.35 s). U18 505 left performance was observed to be slower compared to U16 (2.57 ± 0.12 vs 2.15 ± 0.17 s) whilst there was little difference between the two age groups in the right (2.52 ± 0.13 vs 2.54 ± 0.14 s) [40]. L-run test performance was shown to be greater in U19 RU players compared to their U16 counterparts (6.21–6.33 vs 6.49–6.42 s) [37,38].

**Table 10:** Change of direction performance for age grade rugby union players.

Age group	Position	Playing level	Change of direction 1 (s)	Change of direction 2 (s)
U13	Back-line [34]	Provincial	20.30 ^a^	13.86 ^b^
	Half Back [34]	Provincial	18.39 ^a^	13.28 ^b^
	Loose Forward [34]	Provincial	18.32 ^a^	12.94 ^b^
	Tight Forward [34]	Provincial	19.90 ^a^	13.85 ^b^
U15	All [64]	Club	5.34 ± 0.20 ^c^	
U16	All [37]	School	6.62 ± 0.46 ^d^	
	All [37]	School	6.49 ± 0.34 ^d^	
	All [40]	Academy	2.15 ± 0.17 ^e^	2.54 ± 0.14 ^f^
	All [33]	National	15.43 ± 1.09 ^a^	
	All [31]	National	15.43 ± 1.09 ^a^	
	Back-line [34]	Provincial	17.09 ^a^	13.08 ^b^
	Half Back [34]	Provincial	17.60 ^a^	12.76 ^b^
	Loose Forward [34]	Provincial	18.24 ^a^	13.08 ^b^
	Tight Forward [34]	Provincial	18.91 ^a^	12.01 ^b^
	All [32]	Provincial	15.2 ± 0.9 ^a^	
	Full back [32]	Provincial	14.7 ± 0.5 ^a^	
	Wing [32]	Provincial	15.2 ± 1.5 ^a^	
	Centre [32]	Provincial	14.8 ± 0.5 ^a^	
	Fly half [32]	Provincial	14.7 ± 0.2 ^a^	
	Scrum half [32]	Provincial	14.6 ± 0.5 ^a^	
	Loose forward [32]	Provincial	15.6 ± 0.9 ^a^	
	Lock [32]	Provincial	15.5 ± 0.9 ^a^	
	Hooker [32]	Provincial	15.2 ± 0.8 ^a^	
	Prop [32]	Provincial	15.8 ± 0.7 ^a^	
U17	All [64]	Club	5.08 ± 0.18 ^c^	
U18	All [40]	Academy	2.57 ± 0.12 ^e^	2.52 ± 0.13 ^f^
	All [31]	National	15.36 ± 0.95 ^a^	
	All [33]	National	15.36 ± 0.95 ^a^	
	Back-line [34]	Provincial	16.68 ^a^	11.23 ^b^
	Half Back [34]	Provincial	16.39 ^a^	10.90 ^b^
	Loose Forward [34]	Provincial	17.46 ^a^	11.39 ^b^
	Tight Forward [34]	Provincial	17.57 ^a^	11.93 ^b^
	All [32]	Provincial	15.1 ± 0.8 ^a^	
	Full back [32]	Provincial	15.0 ± 0.7 ^a^	
	Wing [32]	Provincial	14.4 ± 0.4 ^a^	
	Centre [32]	Provincial	14.4 ± 0.2 ^a^	
	Fly half [32]	Provincial	14.5 ± 0.4 ^a^	
	Scrum half [32]	Provincial	15.1 ± 0.3 ^a^	
	Loose forward [32]	Provincial	15.0 ± 0.3 ^a^	
	Lock [32]	Provincial	15.4 ± 0.6 ^a^	
	Hooker [32]	Provincial	14.9 ± 0.5 ^a^	
	Prop [32]	Provincial	16.3 ± 1.2 ^a^	
U19	All [38]	School	6.33 ± 0.33 ^d^	
	All [38]	School	6.21 ± 0.32 ^d^	
	All [64]	Club	5.02 ± 0.35 ^c^	
	Back-line [34]	Provincial	15.71 ^a^	10.73 ^b^
	Half Back [34]	Provincial	15.94 ^a^	10.44 ^b^
	Loose Forward [34]	Provincial	16.29 ^a^	11.05 ^b^
	Tight Forward [34]	Provincial	17.19 ^a^	11.66 ^b^

Data expressed as mean ± SD

^a^ Illinois agility test

^b^ T-test

^c^ Pro agility

^d^ L-run test

^e^ 505 left

^f^ 505 right

When positional differences were accounted for, the back-line were the slowest during the Illinois agility test (20.30 s) and T-test (13.86 s) at U13 [34]. In the same study, Van Gent & Spamer [34] identified the back-line to be fastest for the Illinois at U16 compared to all positions (Table 10). Durandt et al. [32] observed backs to achieve faster times for the Illinois agility test with a range of 14.70 to 14.80 s for U16 and 14.40 to 15.10 s for U18 compared to 15.20 to 15.80 s and 14.90 to 16.30 s for forwards.

#### Aerobic capacity

Four tests reported by fifteen articles were used to quantify the aerobic capacity of U14 to U20 (Table 11). Estimated VO_2_max from the MSFT was similar for U14, U15 and U16 (47.6 ± 4.7, 47.0 ± 6.9 and 44.1 ± 5.7 mL·kg^-1^·min^-1^) while U20’s are reported to have the greatest (53.7 ± 5.1 mL·kg^-1^·min^-1^) [30]. U15 (1385.4 ± 621.3 m) performance in the YYE1 test was lower than both U17 (1851.3 ± 507.4 m) and U19 (1789.2 ± 507.4 m) [64]. As was U16 (1030.7–1144.6 m) performance in the YYIRT1 compared to U18 (1225 ± 378.8 m), which was lower than both U17 (1851.3 ± 507.4 m) and U19 (1443.6–1789.2 m) [37,38,40,64]. Similar 30-15IFT scores were observed between U16 (18.4–18.9 km.h^-1^) and U18 (18.6–19.1 km.h^-1^) [39,73].

**Table 11:** Aerobic performance for age grade rugby union players.

Age group	Position	Playing level	Aerobic test 1	Aerobic test 2
U14	All [35]	School	47.55 ± 4.56 ^a^	
U15	All [35]	School	46.99 ± 6.86 ^a^	
	All [64]	Club	1385.4 ± 621.3 ^b^	
U16	All [35]	School	44.14 ± 5.68 ^a^	
	All [37]	School	1030.7 ± 269.6 ^b^	
	All [37]	School	1307.3 ± 228.6 ^b^	
	All [66]	Academy	1150 ± 403 ^c^	
	All [40]	Academy	1144.6 ± 337.2 ^c^	18.4 ± 1.3 ^d^
	All [73]	Academy	18.9 ± 1.1 ^d^	
	All [47]	National draft camp	51.3 ± 6.4 ^a^	
	Backs [39]	Academy	1346.6 ± 220.6 ^c^	18.8 ± 1.1 ^d^
	Forwards [39]	Academy	971.4 ± 327.7 ^c^	18.0 ± 1.4 ^d^
	All [32]	Provincial	87.1 ± 19.4 ^e^	
	Full back [32]	Provincial	92.0 ± 18.4 ^e^	
	Wing [32]	Provincial	86.6 ± 24.1 ^e^	
	Centre [32]	Provincial	86.8 ± 9.7 ^e^	
	Fly half [32]	Provincial	98.3 ± 13.6 ^e^	
	Scrum half [32]	Provincial	85.7 ± 5.7 ^e^	
	Loose forward [32]	Provincial	97.5 ± 24.0 ^e^	
	Lock [32]	Provincial	89.8 ± 22.6 ^e^	
	Hooker [32]	Provincial	89.0 ± 7.6 ^e^	
	Prop [32]	Provincial	68.1 ± 13.0 ^e^	
U17	All [64]	Club	1851.3 ± 507.4 ^b^	
U18	All [59]	School	1022 ± 515 ^c^	
	All [40]	Academy	1225 ± 373.8 ^c^	18.6 ± 1.1 ^d^
	All [73]	Academy	19.1 ± 1.1 ^d^	
	All [59]	Academy	1245 ± 451 ^c^	
	Backs [39]	Academy	1466.6 ± 450.9 ^c^	19.2 ± 0.98 ^d^
	Forwards [39]	Academy	1080.0 ± 240 ^c^	18.2 ± 1.1 ^d^
	Backs [68]	Academy	2023 ± 197 ^b^	
	Forwards [68]	Academy	1320 ± 362 ^b^	
	All [32]	Provincial	93.5 ± 15.3 ^e^	
	Full back [32]	Provincial	97.0 ± 3.5 ^e^	
	Wing [32]	Provincial	93.0 ± 10.1 ^e^	
	Centre [32]	Provincial	99.9 ± 23.6 ^e^	
	Fly half [32]	Provincial	98.7 ± 14.3 ^e^	
	Scrum half [32]	Provincial	109.8 ± 12.0 ^e^	
	Loose forward [32]	Provincial	94.8 ± 12.8 ^e^	
	Lock [32]	Provincial	90.0 ± 10.6 ^e^	
	Hooker [32]	Provincial	92.8 ± 12.8 ^e^	
	Prop [32]	Provincial	77.6 ± 11.1 ^e^	
U19	All [38]	School	1443.6 ± 259.1 ^b^	
	All [38]	School	1505.9 ± 75.8 ^b^	
	All [64]	Club	1789.2 ± 507.4 ^b^	
	Backs [68]	Academy	1954 ± 321 ^b^	
	Forwards [68]	Academy	1460 ± 320 ^b^	
	Backs [36]	National	50.65 ± 3.76 ^a^	
	Forwards [36]	National	47.08 ± 4.24 ^a^	
U20	All [30]	University	53.7 ± 5.1 ^a^	
	Backs [30]	University	56.3 ± 21.7 ^a^	
	Forwards [30]	University	51.6 ± 14.7 ^a^	
	Backs [68]	Academy	1943 ± 124 ^b^	
	Forwards [68]	Academy	1460 ± 387 ^b^	
	Backs [57]	National	102 ± 12 ^e^	
	Forwards [57]	National	86 ± 15 ^e^	

Data expressed as mean ± SD

^a^ Estimated VO_2_max from multistage fitness test (mL·kg^-1^·min^-1^)

^b^ Yo-yo endurance test level 1 (m)

^c^ Yo-yo intermittent recovery test level 1 (m)

^d^ 30–15 Intermittent Fitness Test (km·h^-1^)

^e^ Stages completed of multistage fitness test (AU)

Differences have been identified between positions with U16, U18 and U19 backs found to run further in the YYIRT1 (1346.6 ± 220.6, 1466.6 ± 450.9 & 1954.0 ± 321.0 m, respectively) and achieve higher finishing velocities in the 30-15IFT (18.8 ± 1.1 & 19.2 ± 0.98 km.h^-1^, respectively) compared to forwards (971.4 ± 327.7, 1080.0 ± 240.0 & 1460.0 ± 320.0 m; 18.0 ± 1.4 & 18.2 ± 1.1 km.h^-1^) [39,68]. Means reported for U19 and U20 backs during the MSFT were superior to forwards, for both estimated VO_2_max at U19 (50.65 ± 3.76 vs 47.08 ± 4.24 mL.kg^-1^.min^-1^) and stages completed at U20 (102 ± 12 vs 86 ± 15 stages) [30,57]. Props completed the least number of stages during the MSFT at U16 (68.1 ± 13.0 stages) and at U18 (77.6 ± 11.1 AU) [32]. In the same test, loose forwards completed the greatest number of stages at U16 (97.5 ± 24.0 stages) while scrum halves completed the highest for U18 (109.8 ± 12.0 stages) [32].

#### Anaerobic endurance

Due to there being no common tests within the literature, anaerobic endurance data was not considered in this section of the review.

## Discussion

This is the first systematic review to outline the testing methods of physical qualities used in age grade RU research and present objective data of the physical qualities and compare between age grade and position. Following the screening process, 42 studies were found to measure physical qualities of age grade (≤ U20) RU players. From these studies, thirty-one were used to quantify the physical qualities of age grade RU players (U11 –U20) within the review, including; anthropometrics, body composition, strength, power, speed, change of direction and aerobic capacity. Although a large proportion of the data was not presented due to the variety in testing methods, more articles were identified to differentiate between age grades and position compared to similar reviews in RL and AFL [24,25]. The majority of research was cross-sectional, included players competing at U16 to U20 and was collected in Australia, Ireland, South Africa and the United Kingdom. It was identified in the review that a large number of tests are used within the literature to quantify physical qualities. When comparing between age grades and positions, differences are apparent and practitioners may find this information useful when evaluating the development of age grade RU players.

### Methods of assessing physical qualities

The review of testing methods in age grade RU identified a total of 70 tests used to measure 7 physical qualities, demonstrating the wide variety of tests used within the research. In the case of power and speed, frequently used tests are easily identified (i.e. VJ, CMJ, 10, 20 30 and 40m). However, this was not the case for other physical qualities (i.e., body fat percentage, strength, change of direction, aerobic and anaerobic endurance). These findings are in accordance with previous literature where it is suggested the range of methods is due to the number of physical qualities important for the sport [21] and the origin of the country of testing [22]. Inconsistencies in the tests used could further be a result of resource constraints such as the availability of technology (e.g. VJ; vert-tec vs. CMJ; force plate or jump mat), time (e.g. 1RM strength testing vs isometric tests) or safety (e.g. 1RM strength testing vs isometric tests in untrained players). Furthermore, a change in the selection of tests over time may be influenced by a greater understanding of RU match demands where tests are selected to be more specific (i.e. sprint distance [14]). Finally, the development of scientific understanding and increased rigour of testing can influence test selection (e.g. validity of press up testing for strength [75]). These factors could be considered to represent a research-practitioner divide, where researchers favour scientific rigour and practitioners speed and cost of assessment. In the future it is therefore important for both practitioners and researchers to work together to design testing batteries to provide useful research and optimise evidence based practice [76]. As previously suggested in RL, there is potential demand for this connection to be made by Governing Bodies to introduce national/league wide testing batteries.

Even if a variety of tests were performed, multiple methods share common output variables. This can occur as a result of different equipment and techniques used for the same test (e.g. jump tests and height). Furthermore, calculations utilising the recorded value can be used to provide a new output variable (e.g. body fat percentage from sum of skinfolds or predicted 1RM from a multiple repetition maximum test). Although this increases the availability of data to be compared and used within practice, caution must be taken when comparing between methods. For example, methods for collecting body fat percentage have previously shown differing degrees of validity in professional RL players [77]. Furthermore, McMahon et al. [78] and Till et al. [79] have suggested it is not possible to confidently compare values between methods, providing specific examples for the CMJ and aerobic testing. In practice comparisons should first be made between studies that have used similar methods before selecting alternative sources.

A common theme throughout the literature is the use of absolute measures of performance with very few articles incorporating relative variables. With the large range of body shapes and sizes observed in RU it is important to consider the role of body size on athletic performance. This is especially important during age grade sport where increases in height and weight are observed during maturation [80]. To account for the effect of body mass, allometric or ratio scaling can be used to normalise strength and power where a larger muscle mass is beneficial [81,82]. In contrast, greater body mass is detrimental for speed performance but contributes to a higher sprint momentum which is shown to relate to collision success in International RU [6]. Although under reported in age grade RU relative measures of 30-15IFT [73] and speed performance [71] have shown to differentiate between age grade and senior RU players where absolute measures did not. Future research should therefore not solely rely on absolute measures of physical qualities but also report values relative to body mass.

### Physical qualities

#### Anthropometric qualities

Anthropometric qualities (height and body mass) are important for RU performance due to the physical nature of the sport [83]. Both height and body mass are shown to increase with age, which is related to the process of growth and maturation [80]. Similar to longitudinal observations in RL, greater differences in anthropometry were found at younger age grades (i.e. U11-16) compared to older age groups as a result of growth and maturation [84,85]. There is a lack of research assessing the relationship between growth, maturation and the development of anthropometric qualities in age grade RU and further research is required to support this. Other factors that may influence a plateau in body mass within older age grades include the chaotic training demands placed on adolescent athletes in combination with low energy intakes compared to energy expenditure [86–88]. Although height and body mass are important for short-term performance it should be noted that Fontana et al. [47] found career success in RU was not dependent on height and body mass at U16. Furthermore, height does not differentiate between Elite U20 and Elite International RU players but there are differences in body mass (93.2 ± 12.3 vs. 102.8 ± 11.9 kg) [71]. It is therefore important to monitor the development of height and body mass throughout age grade RU as players go through maturation and develop towards the professional level.

When comparing between positions, forwards are generally both taller and heavier than backs, which are consistent with findings in senior players [10]. The size and mass of the forwards is better suited to the collision and set piece demands (i.e. scrum and lineout) they endure during a match [17]. Locks, also considered tight forwards, are the tallest at all age groups due to their role in the lineout where the goal is to maximise the peak height of the catch [89]. Furthermore, scrum halves are the lightest and smallest likely relating to the higher locomotive and reduced collision demands during match play in comparison to other positions [3]. Anthropometrics should be considered during the talent identification process and positional selection in combination with growth and maturation status.

#### Body composition

Body composition is an important consideration for performance as excessive body fat can negatively affect physical performance, for example acceleration and the metabolic cost of exercise [10]. Although only one study in the review reported body fat percentage using DXA [54], the gold standard for measuring body composition, all methods reported similar body fat % across age groups suggesting body composition remains reasonably stable. This was similar to cross sectional findings in RL although only skinfold thickness was reported [25]. Interestingly, longitudinal research in RL [90,91] and elite RU [92] has identified improvements in body composition during training periods, however no research has been carried out in age grade RU players to support this. Although similar results are reported for age grade players and elite international players (backs 10.7% and forwards 14.2%) [93], career progression from U16 to international as opposed to other playing levels was differentiated by a lower body fat percentage [47]. The acceptable body composition of age grade RU players is unknown, however further research into the longitudinal change and the interaction with other physical qualities is important for optimising both long and short-term performance.

Although there are limited observed differences between age grades, body composition is different between positions. Tight five and more specifically front row players are identified to have greater body fat than other positions [32,34]. Similar to height and body mass these are likely aligned with match demands with forwards playing fewer minutes, covering less distance and involved in more collisions [11,17].

#### Muscular strength

Muscular strength is important for RU performance due to the collision component of the sport and its relationship with other physical qualities (i.e. power and speed) [94]. This review found absolute muscular strength to be greater in older age grades. The differences observed between age grades is thought to be due to a combination of resistance training experience, and growth and maturation [60]. Similar to AFL [24] and RL [25], there is a paucity of research regarding the strength of age grade RU players, specifically players under the age of 16 years for measurements of lower body and upper body pull muscular strength. This may be a result of low training age where appropriate movement patterns are not yet developed for intense loads [95]. Future research should utilise testing methods which require less technique and therefore a lower injury risk while providing a valid and reliable measure of muscular strength such as the isometric mid-thigh pull previously used by Darrall-Jones et al. [40].

Forwards demonstrate greater muscular strength at all age grades compared to backs. It is favourable for forwards to possess greater absolute strength due to the positional demands placed on forwards performing more collisions, static high intensity efforts and lifting in the lineout [3]. Due to the greater emphasis on sprinting and explosive performance for the backs, relative strength data should be incorporated as the relationship between force production capabilities and body mass is important for optimising performance. Although increasing relative strength is important, its assessment in age grade RU is limited [38,40,60] with no positional comparisons. To further understand the positional differences of age grade RU players both absolute and relative strength measure should be reported.

#### Muscular power

Muscular power is important for success in collisions and the contact element of RU [10]. Similar to changes in height and mass, greater differences at younger age groups (≤ U15) were observed compared to older age groups (U16-U20) for both VJ and CMJ. At older age groups evidence utilising a cross sectional design is conflicting with Darrall-Jones et al. [40] observing differences in CMJ height between U18 and U18 while Kobal et al. [64] observed no difference between U17 and U19. The inconsistency observed in this review is similar to the AFL pathway and is suggested to be a result of the differences in maturity levels [24]. In contrast longitudinal research found increases in jump height during the playing season for U16 [60] and over multiple seasons from U18 to U20 [68]. Longitudinal research may provide a better explanation for the development of muscular power compared to cross sectional research.

Regarding playing position, backs generally have a greater jump height. It should be noted that although greater heights were reported for backs only Spammer & Hattingh [49] identified positional differences of practical significance at U18 and U19. Forwards jump performance may be constrained due to greater body mass and body fat percentage influencing their ability to exert force rapidly. Ball et al. [30] were the only study to use peak power to differentiate between positions with forwards producing greater power outputs. CMJ height may therefore be a poor proxy of muscular power when quantifying positional differences. There is no research to date which reports both direct and indirect measures of power for positional groups to suggest which method is superior. The use of both direct and indirect measures of power may be more appropriate to evaluate and monitor player development compared to jump height alone [40,41].

#### Linear speed

Linear speed is an important physical quality as it is associated with meters made, evasion and line and tackle breaks in senior RU [96]. Additionally, superior speed performance at U16 is suggested to differentiate career progression at the highest level [47]. This review suggests linear speed improves in younger age grades until U16 where further improvements become unclear. Kobal et al. [64] identified differences in 10, 20 and 30m sprint performance between U15 and older U17 and U19 players. No further increases in performance were found in studies that compared players competing at U16 and older [32,39,40]. As a result of growth, the development of longer limbs is suggested to influence stride length and frequency enhancing performance at a young age (< 16 years) before entering the period of peak weight velocity (> 16 years) which limits the development of speed qualities [80]. The constraint of body mass on speed development is supported by Casserly et al. [68] who suggest small increases in body mass act as a mediator of speed development from U18-U20. Furthermore, Barr et al. [71] identified sprint performance does not improve from U20 international to senior international, but rather body mass increases resulting in greater momentum. Both studies are however limited to age groups which are post maturation and only utilise a single team which may not reflect the variety of training interventions used within RU. Further longitudinal research is required to understand the concurrent development of body mass and speed during the earlier stages of growth and maturation.

Backs are shown to be quicker than forwards across a range of age groups and distances [32,36,39,49,57]. This is associated with the positional demands of RU where forwards are exposed to lower running demands while backs are often in open space with greater opportunities to run [97]. Although the differences between backs and forwards are consistent throughout the literature, the limited differences observed between individual positions may be exaggerated by the level of accuracy used by Durant [32], the only author to quantify linear speed performance of individual positions. Potentially due to the large amount of cross-sectional research utilising a small number of teams, positional differences are limited throughout this review as a result of small sample sizes. Future research should attempt to increase sample sizes using multiple clubs to increase the knowledge of positional differences in age grade RU players. Furthermore, due to a greater body mass compared to backs, forwards achieve a greater momentum even though they are observed to be slower [39]. In addition to the monitoring of speed qualities, changes in body mass should also be considered when goal setting and evaluating the development of positional speed qualities.

#### Change of direction

The ability to rapidly change direction is important in RU for match actions such as evasive running when attacking [98]. Findings suggest there are some differences in younger players U13-U16 [34,64], however differences in older players (> U16) are unclear with similar scores observed between age groups [32,34,40,64]. This contrasts findings in RL literature where a general improvement was observed with age, however the availability of change of direction data for older age grade RU players is limited [25]. These differences may be a result of the development of longer limbs during growth prior to an increase in body mass [80]. Increased body mass is thought to negatively effect change of direction ability as it increases the eccentric breaking required to reduce momentum and change direction [99]. This may explain an improvement in 505 results from U18 to U21 which are accompanied by increases in relative strength [40]. While the literature provides an insight of the change of direction ability of age grade RU players, no research was included within this review which assess the agility of RU players, where agility incorporates the response to a stimulus [100]. Future research should not only consider of the development and trainability of the components making up change of direction (i.e. accelerating, decelerating and reaccelerating), but also the ability to perform these actions in response to a stimulus.

Positional differences are less apparent in the research conducted. For both the Illinois agility test and T-test differences were unclear, with the exception of props [32,34]. This is in alignment with the findings in RL, but unlike RL, not all RU players are exposed to regular changes of direction due to defensive retreats [25]. It would therefore be beneficial to gain further understanding of positional specific movement signatures to assist with the development of change of direction ability.

#### Aerobic capacity

A well-developed aerobic capacity is needed for RU to be able to recover between high intensity bouts [19]. Current research is contradictory suggesting aerobic capacity does [32,39] and does not [39,40,64,68,73] differentiate between age grades. The contrast in findings may be due to the differences in tests used with Darrall-Jones et al. [39] identifying U18’s cover a likely greater distance than U16 in the YYIR1, but no difference is observed for the 30-15IFT. Furthermore, when body mass is considered as a covariate differences are observed between age grades for both the 30-15IFT [73] and YYIR1 [68]. These findings suggest increases in body mass may mask expected improvements in aerobic capacity due to training and maturation during adolescents [101]. In contrast to these findings longitudinal research in rugby league has identified seasonal [90,102] and annual [85] improvements in aerobic capacity. Casserly et al. [68] provide the only study to longitudinally monitor the development of aerobic capacity in RU observing no change, however the age groups included were older than the RL players observed and post the effects of maturation. Utilising cross sectional data to assess the differences between age groups may not be appropriate and further longitudinal research may provide further information on the development of aerobic capacity and its relationship with body mass.

Positional differences are observed in the aerobic capacity of backs and forwards [32,36,39,57]. Forwards, more specifically props, are identified to be the worst performers [32]. Similar to linear speed and change of direction ability a greater body mass may influence performance in aerobic tests, especially those that contain a change of direction. The reduced aerobic capacity is associated with the playing demands of forwards who cover less ground and often have a reduced playing time compared to backs [103].

### Limitations

First, a major limitation of the current literature is the diversity of tests used to quantify the physical qualities of age grade RU players. Although some common variables are identified throughout the literature, the lack of homogeneity in testing methods limits the effectiveness of the research when making comparisons between age grades across the review. Secondly, the research is limited by the large number of studies utilising a cross sectional design to compare between age grades. Although comparisons between independent samples provide a snapshot of differences between age grades, inferences cannot be made about the development of physical qualities on an individual level. Thirdly, the research regarding positional specific physical qualities is limited, potentially due to limited sample sizes. It is well documented that positional match demands vary greatly and therefore each will possess a specific set of physical qualities, however only two studies [32,34] report the physical qualities for positional sub groups (e.g., props, hookers, locks or tight forwards) as opposed to a unit (forwards and backs) or full team. Fourthly, there is a paucity of information regarding RU players under the age of 16 years. Understanding physical qualities below the age of 16 could enhance the prescription of physical development to align with an appropriate long-term athlete development programme. Finally, a limitation of this review was the inability to carry out a meta-analysis on the reported data providing summary normative values for the results presented. Due to the variety of tests, methods, positional groups and playing standards reported, no further analysis was carried out.

### Future research

Future studies on the physical qualities of age grade RU players should build upon the current literature by increasing the availability of data using similar physical tests. It may be beneficial for researchers to work alongside practitioners or national governing bodies to develop national standardised testing batteries that are both practical and evidence based. The development of standardised testing batteries could provide the opportunity for studies to recruit samples from multiple clubs, thus increasing samples sizes, generalisability of results and statistical power of subcategory comparisons (e.g. position or playing level). It would also be advantageous to carry out longitudinal research on age grade RU players to better inform the development of physical qualities rather than the differences between two samples. The resultant normative values for changes in physical qualities would enhance the ability of practitioners to set achievable short and long-term goals. Furthermore, statistical modelling should consider the interactions between physical qualities (e.g. body mass and linear speed) and factors which may influence the development of physical qualities (i.e. growth and maturation), which have been discussed as potentially confounding factors within this review, to enhance the ability of practitioners to prescribe holistic training programs in accordance with long term athlete development models. Finally, further research should utilise innovative analysis techniques to increase the understanding of player selection and career progression throughout the talent identification pathway. Developments in data visualisation techniques should accompany this to increase the practicality of the data to all coaches and not just those with a statistical background.

## Conclusion

Provision of normative data for the physical qualities of age grade RU players is important for practitioners to evaluate athlete performance, guide training prescription and inform goal setting. This is the first systematic review to collate the tests used throughout the literature to identify the physical qualities of age grade RU players and present the current evidence by age grade and position. Seventy-five tests were identified to assess seven physical qualities (body composition, muscular strength, muscular power, linear speed, change of direction ability, aerobic capacity and anaerobic endurance capacity). When comparing the physical qualities between age grades differences are apparent between younger age grades (≤ U16). Although older age groups (U19—U20) generally performed the best in physical testing, increased physical performance was not always clear between U16 and U20 age grades, except for muscular strength. The differences at all age groups are potentially due to factors such as increased training exposure and growth and maturation. Relative measures of physical qualities could further distinguish between age grades to account for increases in body mass associated with training, growth and maturation. Positional differences observed are often related to match demands with forwards being taller, heavier and stronger while backs are faster and fitter and therefore training should reflect these differences. The normative data presented in this review can be used by practitioners to evaluate the physical qualities of age grade rugby players and subsequently prescribe appropriate training programmes. The practical use of the data is limited however by the variety of testing methods used, lack of positional data and the paucity of longitudinal research. The use of standardised testing batteries may be beneficial for further research to guide the physical development of age grade RU players.

## Supporting information

S1 File PRISMA checklist. (DOC)
